# Is a Win–Win possible? Achieving pareto-optimal privacy-utility balance in fine-tuned genome language model embeddings against embedding reconstruction attacks

**DOI:** 10.1093/bioinformatics/btag311

**Published:** 2026-07-07

**Authors:** Reem Al-Saidi, Erman Ayday, Ziad Kobti, Rola AlSeidi

**Affiliations:** School of Computer Science, University of Windsor, Windsor, Ontario, N9B 3P4, Canada; Department of Computer and Data Sciences, Case Western Reserve University (CWRU), Ohio, 44106, United States; School of Computer Science, University of Windsor, Windsor, Ontario, N9B 3P4, Canada; Department of Mathematics, Philadelphia University, Amman, 19392, Jordan

## Abstract

**Motivation:**

Genomic data is among the most sensitive categories of personal information, and the growing adoption of language models for sequence analysis raises significant privacy concerns. Prior work demonstrated that embeddings from general-purpose language models adapted for genomic sequences leak substantial single-nucleotide information under reconstruction attacks, and that fine-tuning embeddings can reduce this vulnerability at certain positions. However, three critical questions remain unaddressed: (i) whether privacy-utility tradeoffs are inherent constraints or configuration-dependent phenomena; (ii) whether genomic-specialized models such as DNABERT-base and Nucleotide Transformer exhibit different vulnerabilities than adapted general-purpose models; and (iii) how to statistically validate whether observed privacy improvements represent meaningful gains. Addressing these gaps is essential for guiding model selection in privacy-sensitive genomic applications.

**Results:**

We systematically evaluated 13 transformer architectures, 9 general-purpose and 4 genomic-specialized, under position-specific embedding reconstruction attacks. We assessed the vulnerabilities of both pre-trained and fine-tuned models to the single-nucleotide inference-reconstruction attack using our new metrics, including error-based privacy gain and Pareto dominance scores, and statistically validated the results via paired *t*-tests. XLNet-Large achieved the best observed privacy protection among all evaluated models (+19.5% mean privacy gain) while maintaining competitive prediction performance. General-purpose models outperformed genomic-specialized models in 56% of pairwise comparisons. Tokenization strategy, rather than domain specialization, emerged as the primary determinant of the privacy-utility balance. These findings provide evidence-based guidance for selecting models in privacy-sensitive **short-window** genomic applications. All privacy claims in this work are specific to position-wise embedding reconstruction attacks and do not extend to other privacy risks, such as membership inference or training data extraction, which may respond differently to fine-tuning.

**Availability and implementation:**

The code is publicly available at https://github.com/AnonymousISCBConf/Win-Win-Privacy-Utility-Analysis.

## 1 Introduction

Genomic data is among the most sensitive categories of personal information, encoding disease susceptibility, hereditary traits, and familial relationships (Naveed *et al.* 2015). As healthcare institutions increasingly deploy large language models for genomic analysis within federated learning pipelines, model-as-a-service platforms, and collaborative research environments (Yu *et al.* 2024, Sakai and Lam 2026), a critical vulnerability emerges: the embeddings exchanged in these collaborative environments remain susceptible to reconstruction attacks, where nucleotide-level information can be inferred from dense vector representation. This creates a fundamental question for practitioners: *Can fine-tuned embeddings deliver strong task utility while measurably reducing reconstruction risk, or must deployers accept an inherent tradeoff?*

Pan *et al*. (2020) demonstrated that embeddings from general-purpose language models adapted for genomic sequences leak substantial single-nucleotide information under reconstruction attacks. Al-Saidi *et al*. (2025) extended this analysis to fully fine-tuned models, finding that fine-tuning can enhance privacy protection in specific models. However, their evaluation framework relied on delta-privacy metrics that could not determine whether privacy improvements came at the expense of utility, thereby preventing the identification of configurations that might enhance both dimensions simultaneously. Three critical gaps remain in the current understanding of embedding privacy. First, existing metrics evaluate privacy in isolation, reporting only changes in reconstruction accuracy without jointly considering utility outcomes, leaving unresolved whether privacy-utility trade-offs are inherent constraints or configuration-dependent phenomena. Second, the vulnerability of genomic-specialized language models remains unexplored despite their growing deployment in sensitive applications; it is unclear whether domain-specific pretraining and DNA tokenization confer privacy advantages. Third, current metrics report numerical differences without quantifying effect magnitude or statistical significance, hindering validation of meaningful improvements versus measurement noise.

We address these gaps through systematic evaluation of 13 transformer architectures spanning both general-purpose models, BERT (Devlin *et al.* 2019), GPT-2 (Radford *et al.* 2019), XLNet (Yang *et al.* 2019), RoBERTa (Liu *et al.* 2019), ERNIE 2.0 (Sun *et al.* 2020) (each in Base/Large or Small/Medium variants), and genomic foundation models, Nucleotide Transformer (Dalla-Torre *et al.* 2023), DNABERT-base (Ji *et al.* 2021), DNABERT-2 (Zhou *et al.* 2023), and DNAGPT (Zhang *et al.* 2023). We extract embeddings in both pretrained and fully fine-tuned states, execute position-specific reconstruction attacks (Al-Saidi *et al.* 2025), and introduce extended evaluation metrics: Privacy Gain (PG) captures relative improvement in reconstruction difficulty, while Pareto Dominance Scores (PDS) jointly categorize privacy-utility outcomes, validated through paired *t*-tests with effect-size analysis. We compare genomic-specialized models, designed with domain-specific tokenization and pretraining on DNA sequences, with general-purpose models adapted for genomic tasks in terms of privacy-utility balance using a Cross-Model Privacy Advantage (CPUA). Our findings challenge two common assumptions. First, privacy-utility trade-offs are not inevitable; specific architectures can achieve genuine “Win–Win” outcomes after fine-tuning. XLNet-Large achieves the strongest balance among general-purpose models, with statistically significant privacy gains (+19.5%) and utility improvements (+16.8%). Second, domain specialization alone is insufficient for privacy protection. Among genomic models, Nucleotide Transformer achieves the best balance (+6.0% privacy, +4.0% utility), while DNABERT-base exhibits privacy degradation despite domain-specific pretraining. Architectural features, permutation language modeling, model capacity, and tokenization granularity emerge as primary determinants. This study presents an empirical benchmarking and systematic evaluation framework for privacy-utility tradeoffs in genomic language model embeddings, providing evidence-based architecture selection guidance for short-window genomic tasks. Formal bounds relating architectural properties to reconstruction vulnerability remain an important direction for future theoretical work.

## 2 Preliminaries

### 2.1 Model architectures

We consider two categories of transformer models for genomic sequence analysis: **(i) General-Purpose Models** are pretrained on natural language corpora using self-supervised objectives. *Masked language models* [e.g. BERT (Devlin *et al.* 2019), RoBERTa (Liu *et al.* 2019), ERNIE 2.0 (Sun *et al.* 2020)] use bidirectional attention, where each token attends to all positions, and pretraining requires predicting randomly masked tokens from the surrounding context. *Permutation language models* [e.g. XLNet (Yang *et al.* 2019)] capture bidirectional context without explicit masking by training on all possible token orderings. *Autoregressive models* [e.g. GPT-2 (Radford *et al.* 2019)] use causal attention, in which each token attends only to preceding positions, and pretraining requires predicting the next token. When applied to DNA sequences, these models process nucleotides through their native subword tokenization schemes.**(ii) Genomic-Specialized Models** are pretrained directly on DNA sequences with domain-specific tokenization. These include models using overlapping k-mer tokenization where each token represents *k* consecutive nucleotides with stride one [e.g. DNABERT-base (Ji *et al.* 2021)], byte-pair encoding learned from genomic corpora [e.g. DNABERT-2 (Zhou *et al.* 2023)], non-overlapping k-mer tokenization [e.g. Nucleotide Transformer (Dalla-Torre *et al.* 2023)], and single-nucleotide tokenization [e.g. DNAGPT (Zhang *et al.* 2023)].

### 2.2 Transformer embeddings

Transformer models map input sequences to dense vector representations through stacked self-attention layers. Given an input sequence of *n* tokens, a transformer with *L* layers produces contextualized hidden states $H^{\left(L\right)}=[h_{1}^{\left(L\right)},h_{2}^{\left(L\right)},\ldots ,h_{n}^{\left(L\right)}]\in R^{n\times d}$, where *d* is the hidden dimension.

For sequence-level tasks, a single embedding vector must represent the entire input. **Encoder models** prepend a special [CLS] token to the input and extract its final-layer representation:


(1) $$e_{\text{seq}}=h_{\left[\mathrm{CLS}\right]}^{\left(L\right)}\in R^{d}$$

This token aggregates sequence-level information through bidirectional self-attention. **Autoregressive models** lack a classification token and instead use the final token’s hidden state, which encodes the cumulative sequence through causal attention:


(2) $$e_{\text{seq}}=h_{n}^{\left(L\right)}\in R^{d}$$

To enable position-specific queries on these sequence embeddings, **sinusoidal positional encodings** (Vaswani *et al.* 2017) provide position information through fixed trigonometric functions. For position *i*, the encoding $p_{i}\in R^{d}$ is defined as:


(3) $$p_{i,2k}=\text{sin}\;\left(i/10000^{2k/d}\right)$$

(4) $$p_{i,2k+1}=\text{cos}\;\left(i/10000^{(2k+1)/d}\right)$$

where $p_{i,2k}$ denotes the 2*k*-th coordinate and *d* is the encoding dimension. This formulation allows a single model to generalize across all positions by concatenating the sequence embedding $e_{\text{seq}}$ with the positional encoding $p_{i}$ for any target position *i*.

### 2.3 Full fine-tuning

Fine-tuning adapts a pretrained model to a downstream task by continuing gradient-based optimization on task-specific data (Wu *et al.* 2025). Given a pretrained model with parameters $\theta_{\text{pre}}$ and a task-specific classification head with parameters ϕ, **full fine-tuning** updates all parameters jointly (Tarkiainen 2025):


(5) $$\theta^{*},\phi^{*}=\arg\underset{\theta ,\phi }{\min}\sum_{i=1}^{N}L\left(f_{\phi }(e_{i}(\theta )),y_{i}\right)$$

where *N* is the number of training samples, $e_{i}(\theta )$ is the embedding of sample *i* produced by the transformer with parameters θ, $f_{\phi }$ is the classification head, $y_{i}$ is the label, and L is the task loss.

### 2.4 Downstream task and dataset

We evaluate all models on **splice-site prediction (Akpokiro *et al.***^2022^**)**, which is a binary classification task distinguishing true exon-intron boundary sequences from decoy sequences in the human genome. In this task, the model recognizes specific nucleotide patterns at specific positions, making it an adversarially rigorous privacy setting, since the position-specific information the model encodes precisely what reconstruction attackers target.

We use the **Homo Sapiens Splice Sites Dataset (HS3D)** (Pollastro and Rampone 2002), comprising 33 680 sequences of L=20 nucleotides centered on splice-site boundaries, the standard fixed-length window for splice-site analysis (Pollastro and Rampone 2002). The dataset is partitioned into 31 680 training sequences and 2000 held-out sequences, ensuring no data leakage between the two phases.

## 3 Threat model and attack methodology

### 3.1 Attack definition

*Notation.*

Let $x=(w_{1},w_{2},\ldots ,w_{L})$ denote a genomic sequence of length *L*, where each $w_{i}\in N=\{A,C,G,T\}$ represents a nucleotide. Let $f_{\theta }:x\to e$ denote a language model parameterized by θ that maps sequence x to a *d*-dimensional embedding $e\in R^{d}$. The adversary aims to recover individual nucleotides from the embedding. Formally, for each position i∈{1,…,L}, the adversary constructs a classifier:


(6) $$A_{i}:R^{d}\to N,\;{\hat{w}}_{i}=A_{i}(e)$$

The attack succeeds at position *i* when ${\hat{w}}_{i}=w_{i}$.

### 3.2 Threat model

We consider an *honest-but-curious* adversary operating in scenarios where genomic embeddings are shared while raw sequences remain protected, including: (i) **Embedding-as-a-service APIs:** Cloud providers offering genomic analysis returning only embeddings, not raw sequences, to downstream applications. (ii) **Data breaches:** An attacker compromising an embedding database (lower security than sequence storage) could attempt reconstruction. (iii) **Model auditing:** Third parties verifying model fairness or bias may receive embeddings without sequence access. We assumed that the adversary knows the DNA sequence format (nucleotide alphabet and sequence length) and the embedding extraction configuration (classification or last token layer). Also, the adversary has access to powerful computing resources, including extra storage, memory, and processing power, which are needed for attack training and inference.

- **Same-Domain Auxiliary Data:** The adversary possesses auxiliary sequences from the same domain as the target embeddings, enabling attack classifiers trained on well-matched data.
- **Black-Box Query Access:** The adversary can submit sequences to the target model and receive corresponding embeddings. The adversary cannot access the model weights, gradients, or intermediate-layer activations.
- **Target Embedding Acquisition:** The adversary obtains embeddings of private victim sequences (e.g. as an honest-but-curious cloud provider, or through a data breach).
- **Model State Knowledge:** The adversary knows whether target embeddings originate from a pretrained or fine-tuned model and has query access to a model in the *same state*.
- **Collusion Access:** This is an *evaluation assumption* only, ground-truth sequences are used solely to measure attack accuracy ${\text{Acc}}_{i}$. Collusion is not a capability exercised by the attacker during inference.

### 3.3 Attack methodology

Following Pan *et al.* (2020) and Al-Saidi *et al.* (2025), we implement the reconstruction attack as position-specific supervised classification. Algorithm 1 presents a high-level description of the attack methodology applied during both the training and testing phases.

Algorithm 1 Embedding Reconstruction Attack.

**Require:** Query access to model $f_{\theta }$, auxiliary sequences $D_{\text{aux}}$, target embeddings $\left\{e_{k}^{*}\right\}$

**Ensure:** Predicted sequences $\left\{{\hat{x}}_{k}\right\}$

**  Phase A: Attack Training** 

1: ** for**$x^{\left(j\right)}\in D_{\text{aux}}$**do** 

2: **  **$e^{\left(j\right)}\leftarrow f_{\theta }(x^{\left(j\right)})$      ▹ Query model

3: ** end for** 

4: ** for** position i=1 to *L* **do** 

5: **  **$T_{i}\leftarrow {\left\{([e^{\left(j\right)}\;⊕\;\text{PE}(i)],w_{i}^{\left(j\right)})\right\}}_{j=1}^{\left|D_{\text{aux}}\right|}$

6: **  **Train classifier $A_{i}$ on $T_{i}$ via cross-entropy

7: ** end for** 

**  Phase B: Attack Inference** 

8: ** for** each target embedding $e_{k}^{*}$do 

9: **  for** position i=1 to *L* **do** 

10: **   **${\hat{w}}_{k,i}\leftarrow A_{i}([e_{k}^{*}⊕\text{PE}(i)])$

11: ** end for** 

12:  ${\hat{x}}_{k}\leftarrow ({\hat{w}}_{k,1},\ldots ,{\hat{w}}_{k,L})$

13: ** end for** 

The positional encoding (PE) concatenation enables position-specific prediction:


(7) $$z_{i}^{\left(j\right)}=[e^{\left(j\right)}\;⊕\;\text{PE}(i)]\in R^{2d}$$

## 4 Evaluation metrics

*Notation:*

We denote pretrained model states as **PT** and fine-tuned model states as **FT**; superscripts PT and FT on accuracy terms follow accordingly throughout this paper.

### 4.1 Metrics from prior work

1. *Reconstruction Accuracy (Pan et al. 2020)*:
  (8) Acci=1|Dtest|∑j∈Dtest╟[w^i(j)=wi(j)]
  where $D_{\text{test}}$ is the attack evaluation set, $w_{i}^{\left(j\right)}$ is the true nucleotide at position *i* in sequence *j*, ${\hat{w}}_{i}^{\left(j\right)}$ is the predicted nucleotide, and ╟[·] is the indicator function.
2. *Delta Privacy (* Δ *P) (Al-Saidi et al. 2025)*:

Absolute change in reconstruction accuracy:


(9) $$\Delta P_{i}={\text{Acc}}_{i}^{\text{PT}}-{\text{Acc}}_{i}^{\text{FT}}$$

Positive values indicate privacy improvement (lower attack accuracy after fine-tuning).

### 4.2 Extended evaluation metrics

1. *Delta Utility (* Δ *U)*:
  Change in downstream task accuracy:
  (10) $$\Delta U={\text{Acc}}_{\text{task}}^{\text{FT}}-{\text{Acc}}_{\text{task}}^{\text{PT}}$$
  Positive values indicate utility improvement after fine-tuning.
2. *Privacy Gain (PG)*.
  Log-ratio of reconstruction errors quantifying *relative* privacy improvement:
  (11) $${\text{PG}}_{i}=\ln\left(\frac{1-{\text{Acc}}_{i}^{\text{FT}}}{1-{\text{Acc}}_{i}^{\text{PT}}}\right)$$
  Positive values indicate improved privacy. Here, (1−Acc) represents the attacker’s error rate, i.e. the probability that reconstruction fails. Positive PG indicates fine-tuning increased attacker errors (improved privacy). When ${\text{Acc}}_{i}^{\text{PT}}=1.0$, we apply ε-clipping ($\varepsilon =10^{-6}$) to avoid a zero denominator; this affects only positions 1 and 20 of BERT-Base and does not materially change aggregate results.
*Average Privacy Gain*.
  (12) $$\bar{\text{PG}}=\frac{1}{L}\sum_{i=1}^{L}{\text{PG}}_{i}$$
  We test whether the mean privacy gain differs from zero via a one-sample *t*-test:
  (13) $$t=\frac{\bar{\text{PG}}}{\text{SE}(\bar{\text{PG}})},\;\text{SE}=\frac{s_{\text{PG}}}{\sqrt{L}},\;df=L-1=19$$
  At α=0.05, the critical value is |t|>2.093. SE is the Standard Error of the mean, and SPG is the Standard Deviation of the Privacy Gain.
3. *Pareto Dominance Score based on (* Δ *P) (PDS-Delta)*.
  Uses binary thresholds for direct comparison: +2 (Win–Win) when both privacy and utility improve; −2 (Lose-Lose) when both degrade; +1 for partial improvement in one dimension; and −1 for any tradeoff where one dimension improves at the expense of the other.
4. *Pareto Dominant Score based on relative privacy change (PG) (PDS-Gain)*.
  Uses graduated thresholds to identify meaningful privacy improvements: $\tau_{s}=0.095$ (≈10% attacker error increase, ln(1.10)≈0.095) and $\tau_{m}=0.049$ (≈5% error increase, ln(1.05)≈0.049). (Thresholds $\tau_{s}$, $\tau_{m}$ derive from log-ratio interpretation, calibrated to exceed training variance and yield meaningful accuracy reduction. These quantify reconstruction difficulty, not formal privacy guarantees.) Scores reflect joint privacy-utility outcomes: +2 (Strong Win–Win) when PG $>\tau_{s}$ and ΔU >0; +1 (Privacy-Win) when $\tau_{m}<$ PG $\leq \tau_{s}$ and ΔU >0; −2 (Lose-Lose) when both degrade; −1 (Tradeoff) otherwise.
*Aggregate PDS-Gain*.
  (14) $$\bar{\text{PDS-Gain}}=\frac{1}{L}\sum_{i=1}^{L}{\text{PDS-Gain}}_{i}\in [-2,+2]$$
5. *Win–Win Percentage (WWP)*:
  Proportion of positions achieving simultaneous improvement:
  (15) WWP=100L∑i=1L╟[PDS-Gaini=+2]
6. *Cross-Model Privacy Utility Advantages (CPUA)*:
  To compare holistic privacy-utility performance between genomic and general-purpose models, we define:
  (16) $${\text{CPUA}}_{j,k}={\bar{\text{PDS-Gain}}}_{j}-{\bar{\text{PDS-Gain}}}_{k}$$

where $M_{j}$ is a genomic model and $M_{k}$ is a general-purpose model, and $\bar{\text{PDS-Gain}}$ represents the mean PDS-Gain across all 20 sequence positions. Positive values indicate genomic models achieve a superior privacy-utility balance through domain-specific architectural optimizations, while negative values indicate the opposite. Those metrics serve complementary roles: Δ**P** reports absolute accuracy change for comparability with prior work (Pan *et al.* 2020, Al-Saidi *et al.* 2025); **PG** operates on the attacker’s error rate, capturing relative improvement across architecturally diverse baselines; **PDS-Gain** applies calibrated thresholds ($\tau_{s}$, $\tau_{m}$) to distinguish meaningful from marginal gains, with **PDS-Delta** as a secondary robustness check; and **CPUA** serves cross-model comparison exclusively.

### 4.3 Attack scope and limitations

We describe three key experimental design choices, their justifications, and their scope limitations.

- **Fixed Sequence Length (** L=20 **):** HS3D provides fixed-length (20bp windows) representation for splice-site analysis (Pollastro and Rampone 2002), enabling controlled cross-model comparisons, direct replication of prior studies (Pan *et al*. 2020, Al-Saidi *et al*. 2025).
- **Single Downstream Task:** We evaluate on splice-site prediction only. Fine-tuning for this task may implicitly discard fine-grained nucleotide identity at non-motif positions if coarser motif-level features suffice; whether this pattern generalizes to other tasks depends on each task’s positional information requirements.
- **Attack Architecture:** We use a 3-layer MLP per position, following Pan *et al*. (2020). This is the architecturally appropriate choice for position-*independent* 4-class prediction, achieving 40%–60% accuracy on vulnerable models versus 25% random baseline, confirming effective extraction. However, because inter-position dependencies are not exploited, reported privacy gains represent *lower bounds*: a stronger sequence-aware adversary could narrow or eliminate them.

## 5 Results and analysis

### 5.1 Privacy gains across models

We extract embeddings using CLS token embeddings for bidirectional encoders and the last token for autoregressive/permutation models. Each classifier $A_{i}$ is a 3-layer MLP (200 hidden units, sigmoid activations, batch normalization) trained with Adam (lr =0.001) for 5 epochs. We evaluate position-specific privacy changes across all 13 transformer architectures after fine-tuning for splice site prediction. The fine-tuning follows the established practice for the transformer fine-tuning (Ji *et al.* 2021, Zhou *et al.* 2023), and attack classifiers followed Pan *et al.* (2020). Supplementary D, available as supplementary data at *Bioinformatics* online confirms stable convergence within these configurations across all 13 models. Table 1 summarizes the average of reconstruction accuracy, privacy changes, and error-based privacy gains.

**Table 1:** Privacy gains across 13 transformer architectures.^a^^,^^b^

Category	Model	Pre Acc	FT Acc	Δ **privacy**	Privacy gain	Positions improved
*General-Purpose*	BERT-Base	0.379	0.360	+1.9%	−1.3%	4/20 (20%)
	BERT-Large	0.394	0.270	+12.3%	+11.3%	17/20 (85%)
	RoBERTa-Base	0.266	0.329	−6.3%	−8.4%	3/20 (15%)
	RoBERTa-Large	0.313	0.344	−3.1%	−5.4%	3/20 (15%)
	GPT2-Small	0.450	0.436	+1.4%	+3.5%	9/20 (45%)
	GPT2-Medium	0.374	0.461	−8.7%	−16.8%	3/20 (15%)
	XLNet-Base	0.470	0.398	+7.1%	+10.1%	15/20 (75%)
	XLNet-Large	0.457	0.316	+14.1%	+20.2%	20/20 (100%)
	ERNIE 2.0	0.250	0.246	+0.4%	+0.5%	11/20 (55%)
*Genomic*	Nucleotide Trans.	0.344	0.305	+3.9%	+6.0%	11/20 (55%)
	DNABERT-base	0.258	0.257	+0.2%	+0.2%	12/20 (60%)
	DNABERT-2	0.275	0.305	−3.0%	−4.6%	7/20 (35%)
	DNAGPT	0.284	0.259	+2.5%	+3.5%	14/20 (70%)

a Privacy Gain is computed using the error-based metric defined in Section 4. Positions Improved indicates the number of sequence positions showing reduced reconstruction accuracy after fine-tuning.

b Positive ΔPrivacy and Privacy Gain values indicate improved privacy (reduced reconstruction vulnerability). Pre Acc and FT Acc denote average reconstruction accuracy across 20 positions for pretrained and fine-tuned embeddings, respectively.

*Key findings:*

**(i) XLNet-Large achieves optimal privacy protection:** With +20.2% average privacy gain and improvements at all 20 positions, XLNet-Large demonstrates that permutation language modeling combined with large model capacity enables superior privacy preservation. XLNet-Large’s privacy advantage likely stems from two complementary hypotheses: permutation language modeling trains over all possible token orderings, discouraging fixed positional mappings and distributing nucleotide-position information across many embedding dimensions; and large capacity combined with permutation pretraining may converge to representations encoding splice-site motifs (GT/AG) without preserving fine-grained nucleotide identity at non-motif positions. Confirming both hypotheses requires future analysis of attention distributions and embedding geometry.**(ii) Capacity scaling effects are architecture-dependent:** BERT-Large substantially improves over BERT-Base (+11.3% versus −1.3% privacy gain), demonstrating positive scaling in bidirectional masked language models. Conversely, GPT2-Medium degrades compared to GPT2-Small (−16.8% versus +3.5%), revealing that capacity expansion in standard autoregressive models exacerbates privacy vulnerabilities rather than mitigating them. **(iii) RoBERTa exhibits consistent privacy degradation:** Both RoBERTa-Base (−8.4%) and RoBERTa-Large (−5.4%) show significant privacy loss, with only 3 of 20 positions improving in each case. This indicates that byte-level BPE tokenization with dynamic masking creates systematic privacy vulnerabilities during fine-tuning that persist regardless of model capacity. **(iv) Genomic specialization alone is insufficient for privacy preservation:** Among genomic models, Nucleotide Transformer achieves the best privacy gain (+6.0%), while DNABERT-base (+0.2%) and DNABERT-2 (−4.6%) show limited or negative gains despite domain-specific pretraining. The poor performance of DNABERT-base and DNABERT-2 is largely a structural artifact of their tokenization schemes: DNABERT-base’s stride-1 overlapping scheme (k=6) encodes 6 consecutive nucleotides into a single token, making local sequence information directly recoverable from that token alone. DNABERT-2’s BPE scheme similarly bundles multiple nucleotides per token, exposing local sequence details to the reconstruction attacker. These vulnerabilities arise from the tokenization design, not from learned architectural properties. This challenges the assumption that genomic-specialized models inherently provide better privacy protection than general-purpose alternatives. **(v) Tokenization strategy is a primary determinant of privacy-utility balance:** Models using finer-grained tokenization consistently achieves stronger privacy profiles: single-nucleotide tokenization (DNAGPT: +3.5%) and non-overlapping k-mer tokenization (Nucleotide Transformer: +6.0%, where each token covers a non-overlapping chunk) both outperform overlapping k-mer models (DNABERT-base: +0.2%, DNABERT-2: −4.6%), confirming that tokenization strategy drives privacy outcomes more strongly than domain-specific pretraining.

### 5.2 Win–Win configurations and statistical validation

We characterize privacy-utility relationships using Pareto Dominance Scores (PDS) under two evaluation criteria: PDS-Delta (any positive change) and PDS-Gain (threshold-based, requiring >10% error reduction for Win–Win classification). Table 2 presents Win–Win percentages alongside statistical validation of privacy and utility improvements.

**Table 2:** Win–Win configurations and statistical significance across 13 transformer architectures.^a^^,^^b^

		**Win–Win %**		**Avg PDS score**		**Privacy gain**		**Utility gain**		
Category	Model	Delta	Gain	Delta	Gain	Mean	t-stat	Mean	*t*-stat	Configuration
*General-Purpose*	BERT-Base	35%	20%	+0.05	−0.20	−0.15%	−0.27	+8.2%	$11.71^{***}$	Utility-Dom
	BERT-Large	80%	40%	+1.40	+0.75	+12.8%	$13.20^{***}$	+15.6%	$18.35^{***}$	Dual-Imp
	RoBERTa-Base	15%	10%	−0.55	−0.65	−8.5%	$-7.24^{***}$	+9.2%	$10.82^{***}$	Tradeoff
	RoBERTa-Large	15%	10%	−0.55	−0.60	−7.9%	$-6.89^{***}$	+9.8%	$11.15^{***}$	Tradeoff
	GPT2-Small	45%	30%	+0.35	+0.15	+3.8%	$3.25^{**}$	+9.5%	$11.59^{***}$	Dual-Imp
	GPT2-Medium	15%	5%	−0.55	−0.70	−8.7%	$-7.91^{***}$	+7.3%	$10.00^{***}$	Tradeoff
	XLNet-Base	60%	45%	+0.80	+0.55	+7.8%	$6.85^{***}$	+12.1%	$12.94^{***}$	Dual-Imp
	XLNet-Large	95%	75%	+1.85	+1.65	+19.5%	$16.42^{***}$	+16.8%	$17.86^{***}$	**Dual-Imp** ^c^
	ERNIE 2.0	55%	10%	+0.65	−0.15	+2.1%	1.46	+11.2%	$12.04^{***}$	Utility-Dom
*Genomic*	Nucleotide Trans.	55%	35%	+0.65	+0.40	+6.7%	$5.63^{***}$	+11.4%	$12.53^{***}$	Dual-Imp
	DNABERT-base	0%	0%	+0.15	−0.70	−9.8%	$-7.72^{***}$	+6.4%	$9.55^{***}$	Tradeoff
	DNABERT-2	35%	10%	+0.05	−0.40	−4.2%	$-3.04^{**}$	+8.9%	$11.27^{***}$	Tradeoff
	DNAGPT	70%	15%	+1.10	+0.15	+10.5%	$9.63^{***}$	+13.8%	$15.68^{***}$	Dual-Imp

a Win–Win positions achieve simultaneous privacy and utility improvements. PDS-Delta uses any positive change; PDS-Gain requires >10% privacy error reduction. Statistical significance evaluated via paired t-tests (df=19, α=0.05, critical |t|>2.093).

b Dual-Imp = Dual Improvement (both privacy and utility significantly improve); Tradeoff = significant privacy degradation with utility gain; Utility-Dom = utility improvement with non-significant privacy change.

c Champion model (highest privacy-utility balance).

∗∗∗ *P* < .001,

∗∗ *P* < .01,

*Key findings:*

**(i) Six models achieve statistically validated dual improvements.** XLNet-Large ($t_{\mathrm{priv}}=16.42$, $t_{\mathrm{util}}=17.86$), BERT-Large ($t_{\mathrm{priv}}=13.20$, $t_{\mathrm{util}}=18.35$), XLNet-Base ($t_{\mathrm{priv}}=6.85$, $t_{\mathrm{util}}=12.94$), DNAGPT ($t_{\mathrm{priv}}=9.63$, $t_{\mathrm{util}}=15.68$), Nucleotide Transformer ($t_{\mathrm{priv}}=5.63$, $t_{\mathrm{util}}=12.53$), and GPT2-Small ($t_{\mathrm{priv}}=3.25$, $t_{\mathrm{util}}=11.59$) demonstrate significant improvements in both dimensions, establishing that privacy-utility tradeoffs are configuration-dependent rather than universal. **(ii) XLNet-Large achieves optimal Win–Win coverage.** With 95%/75% Win–Win positions under PDS-Delta/PDS-Gain and the highest privacy t-statistic (t=16.42), XLNet-Large represents the champion configuration. Permutation language modeling combined with large capacity enables near-universal dual optimization, with only position 14 exhibiting tradeoff patterns. **(iii) Metric divergence reveals threshold-proximity effects.** ERNIE 2.0 exhibits extreme divergence (55% versus 10% Win–Win), with privacy improvements failing statistical validation (t=1.46, p=0.161). Similarly, DNAGPT shows 70% versus 15% Win–Win, indicating improvements approach but often fail to exceed PDS-Gain’s strict 10% threshold despite achieving statistical significance.**(iv) Five models exhibit statistically validated privacy degradation.** RoBERTa-Base (t=−7.24), RoBERTa-Large (t=−6.89), GPT2-Medium (t=−7.91), DNABERT-base (t=−7.72), and DNABERT-2 (t=−3.04) show significant privacy loss during fine-tuning, confirming that certain architectural configurations create systematic privacy-utility tradeoffs. **(v) Capacity scaling effects diverge by architecture.** BERT-Large dramatically improves over BERT-Base (80% versus 35% Win–Win; $t_{\mathrm{priv}}$: 13.20 versus –0.27), while GPT2-Medium catastrophically degrades compared to GPT2-Small (15% versus 45% Win–Win; $t_{\mathrm{priv}}$: −7.91 versus 3.25). This establishes that scaling benefits depend critically on architectural design rather than parameter count alone. **(vi) Genomic specialization does not guarantee favorable outcomes.** DNABERT-base achieves 0% Win–Win under both metrics with severe privacy degradation (t=−7.72), while the general-purpose XLNet-Large achieves 95%/75% Win–Win. Among genomic models, only DNAGPT and Nucleotide Transformer achieve dual improvements, with tokenization strategy (nucleotide-level versus k-mer) emerging as the critical differentiator.

To verify that Win–Win findings are not an artifact of including architecturally incompatible models, we note that three models show near-chance absolute accuracy on splice-site prediction (Supplementary C, available as supplementary data at *Bioinformatics* online): DNABERT-base (50.0%), ERNIE 2.0 (52.5%), and DNAGPT (52.9%). Importantly, neither DNABERT-base nor ERNIE 2.0 contribute to Win–Win claims, they are already classified as Tradeoff and Utility-Dom, respectively, in Table 2. DNAGPT does achieve statistically significant dual improvements ($t_{\text{priv}}=9.63$, $t_{\text{util}}=15.68$); however, given its near-chance task accuracy, these gains likely reflect low task engagement rather than genuine privacy-preserving representation learning, and should be interpreted accordingly. The remaining five dual-improvement models: XLNet-Large, BERT-Large, XLNet-Base, Nucleotide Transformer, and GPT2-Small. All achieve accuracy between 61.4% and 66.1% (Supplementary C, available as supplementary data at *Bioinformatics* online), confirming that the Win–Win phenomenon holds robustly within architecturally competitive configurations.

### 5.3 Cross model comparison

**Figure 1:**
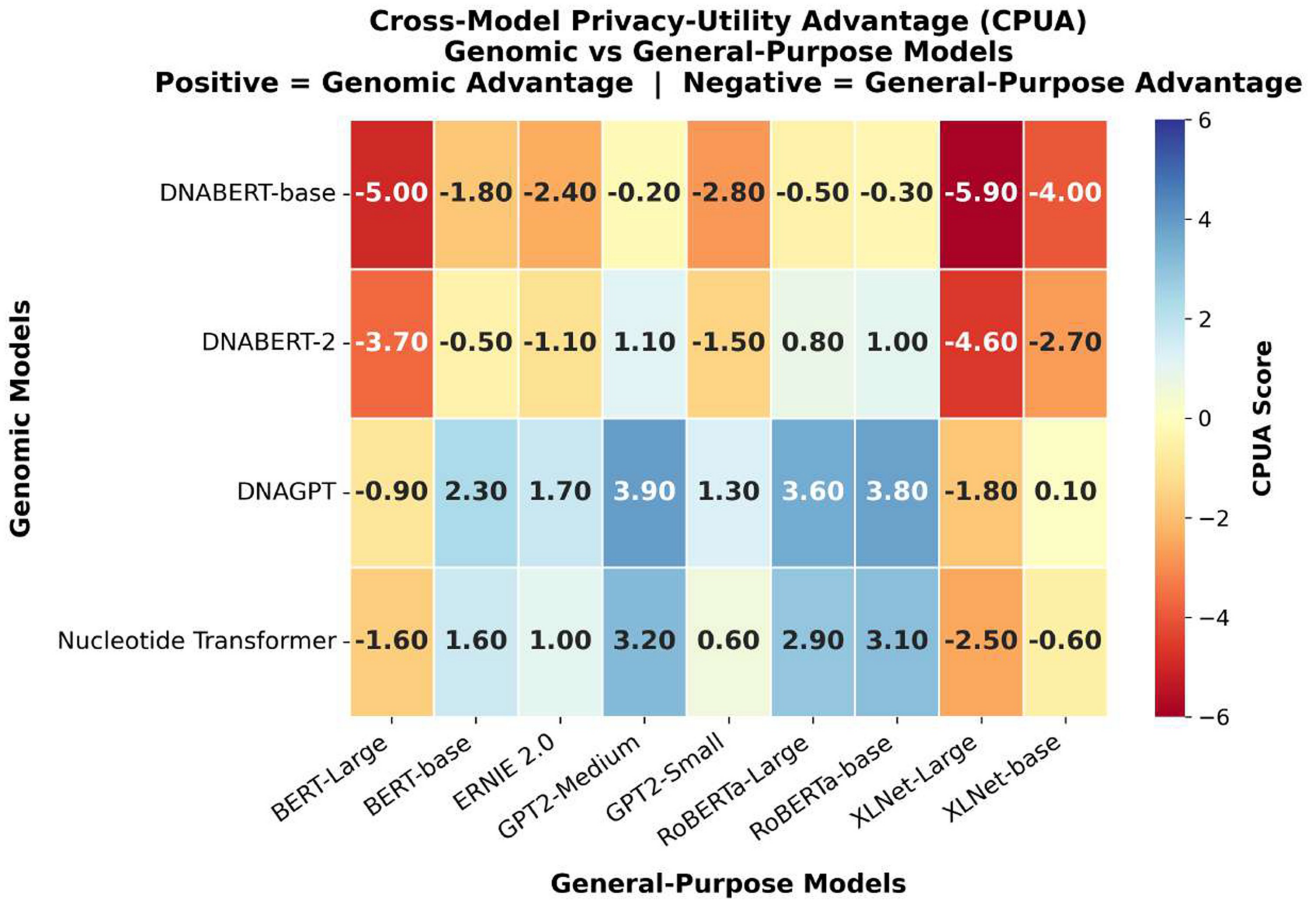
Cross-Model Privacy-Utility Advantage (CPUA) comparing genomic versus general-purpose models. Blue cells indicate genomic model advantage; red/orange cells indicate general-purpose advantage; color intensity reflects score magnitude. XLNet-Large dominates all genomic models (rightmost column, CPUA: −1.80 to −5.90), while DNAGPT emerges as the strongest genomic architecture (third row, CPUA: −1.80 to +3.90).

Figure 1 presents pairwise Cross-Model Privacy-Utility Advantage (CPUA) comparisons between four genomic and nine general-purpose models.

Clear performance tiers emerge: XLNet-Large dominates all genomic models (CPUA: −1.80 to −5.90), while DNAGPT outperforms weaker general-purpose models including GPT2-Medium (+3.90) and RoBERTa-base (+3.80). DNABERT-base shows a systematic disadvantage against all nine general-purpose models. Two factors primarily determine cross-model performance. First, *tokenization strategy supersedes domain specialization*: the 4.22 CPUA-point gap between nucleotide-level tokenization (DNAGPT, Nucleotide Transformer) and k-mer tokenization (DNA BERT base) exceeds the range among general-purpose models. Second, *capacity scaling is architecture-conditional*: bidirectional models benefit from scaling (BERT-Large outperforms BERT-base by +3.20 CPUA), whereas autoregressive models exhibit negative scaling (GPT2-Medium underperforms GPT2-Small by −2.60 CPUA).

## 6. Conclusion and future work

We presented a systematic evaluation of privacy-utility tradeoffs in genomic language model embeddings under position-wise embedding reconstruction attacks, evaluated on fixed-length 20-nucleotide splice-site sequences across 13 transformer architectures. Our findings apply directly to short-window, position-sensitive genomic tasks, and should be interpreted as reconstruction-attack-specific rather than as general privacy improvements from fine-tuning.

Our analysis yields three principal findings. First, privacy-utility trade-offs are configuration-dependent rather than universal constraints: six models achieve statistically validated dual improvements, refuting the assumption that privacy enhancement inevitably sacrifices downstream performance. Second, XLNet-Large emerges as the champion configuration, achieving 95% Win–Win position coverage and demonstrating that permutation language modeling combined with large capacity enables near-universal dual optimization. Third, genomic specialization alone is insufficient for a favorable privacy-utility balance; general-purpose models consistently outperformed DNABERT-base variants, with the tokenization strategy (nucleotide-level versus k-mer) identified as the critical architectural differentiator rather than domain-specific pretraining.

Several extensions merit investigation: (i) evaluating on larger-scale genomic datasets and diverse tasks (promoter prediction, variant effect classification) to establish generalizability across sequence lengths; (ii) characterizing alternative attack vectors, including gradient-based and membership inference attacks; and (iii) developing formal bounds relating architectural properties (attention mechanism, tokenization granularity) to reconstruction vulnerability, which would enable principled privacy-preserving model design.

## Supplementary Material

btag311_Supplementary_Data
