# Supplementary material for: Is a Win–Win possible? Achieving pareto-optimal privacy-utility balance in fine-tuned genome language model embeddings against embedding reconstruction attacks
Source: Bioinformatics. 2026 Jul 7;42(Suppl 1):btag311. doi: 10.1093/bioinformatics/btag311 (PMC13340166; doi:10.1093/bioinformatics/btag311)
Supplement: btag311_Supplementary_Data [file btag311_supplementary_data.pdf]

Supplementary Materials for: Is a Win-Win Possible? Achieving Pareto-Optimal Privacy-Utility Balance in Fine-Tuned Genome Language Model Embeddings Against Embedding Reconstruction Attacks

Contents

1 Supplementary A: Reconstruction Attack Accuracy Tables 3

2 Supplementary B: Position-Specific Privacy Gain Profiles Across All 13 Models 6

2.1 B.1 General-Purpose Models . . . 6
2.1.1 B.1.1 BERT-Base . . . 6
2.1.2 B.1.2 BERT-Large . . . 7
2.1.3 B.1.3 RoBERTa-Base . . . 8
2.1.4 B.1.4 RoBERTa-Large . . . 9
2.1.5 B.1.5 GPT2-Small . . . 10
2.1.6 B.1.6 GPT2-Medium . . . 11
2.1.7 B.1.7 ERNIE 2.0 . . . 12
2.1.8 B.1.8 XLNet-Base . . . 13
2.1.9 B.1.9 XLNet-Large . . . 14
2.2 B.2 Genomic Foundation Models . . . 15
2.2.1 B.2.1 DNABERT . . . 15
2.2.2 B.2.2 DNABERT-2 . . . 16
2.2.3 B.2.3 DNAGPT . . . 17
2.2.4 B.2.4 Nucleotide Transformer . . . 18

3 Supplementary C: Position-Specific Privacy-Utility Analysis 18

3.1 C.1 General-Purpose Models . . . 18
3.1.1 C.1.1 BERT-Base . . . 18
3.1.2 C.1.2 BERT-Large . . . 19
3.1.3 C.1.3 RoBERTa-Base . . . 20
3.1.4 C.1.4 RoBERTa-Large . . . 21
3.1.5 C.1.5 XLNet-Base . . . 22
3.1.6 C.1.6 XLNet-Large . . . 23
3.1.7 C.1.7 GPT2-Small . . . 24
3.1.8 C.1.8 GPT2-Medium . . . 25
3.1.9 C.1.9 ERNIE 2.0 . . . 26
3.2 C.2 Genomic Foundation Models . . . 27
3.2.1 C.2.1 DNAGPT . . . 27

3.2.2 C.2.2 Nucleotide Transformer . . . 28
3.2.3 C.2.3 DNABERT-Base . . . 29
3.2.4 C.2.4 DNABERT-2 . . . 30

4 Supplementary D: Fine-Tuning Evaluation Metrics **32**
4.1 D.1 Fine-Tuning Configuration . . . 32
4.1.1 General-Purpose Models . . . 32
4.1.2 Genomic-Specialized Models . . . 36

5 Supplementary E :Statistical Significance Testing: Comprehensive Analysis **39**
5.1 E.1 Methodology and Assumptions . . . 39
5.2 E.2 Normality Verification . . . 40
5.2.1 Shapiro-Wilk Test Results . . . 40
5.3 E.3 Complete Statistical Results . . . 41
5.3.1 General-Purpose Models . . . 41
5.3.2 Genomic Foundation Models . . . 46
5.4 E.4 Summary . . . 47

1 Supplementary A: Reconstruction Attack Accuracy Tables

This section presents complete position-specific reconstruction attack accuracy results for all 13 evaluated transformer models. For general-purpose models (BERT-Base/Large, GPT-2 Small/Medium, XLNet-Base/Large, RoBERTa-Base/Large, and ERNIE 2.0), we include comparisons with prior work by Pan et al. [2] and Al-Saidi et al. [1] where available. For genomic-specialized models (DNABERT, DNABERT-2, DNAGPT, and Nucleotide Transformer), we report results from our evaluation framework.

Table 1: Reconstruction Attack Accuracy for General-Purpose Transformer Models: Comparison with Pan et al. [2] and Al-Saidi et al. [1]

**Model**	**Embedding**	**P1**	**P2**	**P3**	**P4**	**P5**	**P6**	**P7**	**P8**	**P9**	**P10**	**P11**	**P12**	**P13**	**P14**	**P15**	**P16**	**P17**	**P18**	**P19**	**P20**	**Avg**
**BERT-Base**	**Pretrained (Ours)**	1.00	.450	.300	.240	.280	.260	.280	.310	.370	.320	.260	.220	.220	.290	.270	.310	.220	.280	.740	1.00	**.379**
	**Finetuned (Ours)**	.450	.430	.400	.340	.310	.250	.320	.310	.260	.290	.340	.390	.360	.380	.400	.300	.390	.420	.470	.440	**.360**
	Pretrained (Al-Saidi)	1.00	.450	.300	.320	.300	.270	.280	.320	.320	.250	.250	.250	.280	.360	.300	.300	.370	.300	.740	1.00	**.380**
	Finetuned (Al-Saidi)	.250	.300	.240	.310	.270	.310	.270	.260	.260	.260	.340	.340	.350	.350	.280	.280	.300	.290	.230	.280	**.302**
	Pan's Ref	.982	.910	.720	.530	.490	.485	.480	.475	.470	.470	.470	.470	.485	.495	.520	.570	.650	.770	.920	–	**.598**
	Pretrained nearly identical (0.379 ours vs 0.380), but our finetuned shows better privacy (0.360 vs 0.302).																					
**BERT-Large**	**Pretrained (Ours)**	.960	.290	.500	.290	.390	.330	.290	.340	.220	.320	.360	.300	.290	.270	.220	.360	.310	.220	.660	1.00	**.394**
	**Finetuned (Ours)**	.260	.280	.320	.230	.260	.270	.260	.280	.260	.240	.250	.300	.280	.260	.290	.270	.310	.290	.280	.220	**.270**
	Pretrained (Al-Saidi)	.760	.300	.300	.320	.310	.360	.290	.310	.290	.280	.290	.280	.330	.350	.390	.390	.400	.620	.180	.970	**.383**
	Finetuned (Al-Saidi)	.330	.340	.280	.360	.250	.350	.240	.230	.380	.310	.350	.290	.280	.290	.350	.310	.310	.290	.280	.260	**.314**
	Pan's Ref	.970	.940	.750	.570	.460	.420	.450	.390	.380	.330	.330	.350	.390	.400	.490	–	–	–	–	–	**–**
	Our finetuned shows SUPERIOR privacy (0.270 vs 0.314). Our 12.4% privacy improvement vs 6.9%.																					
**XLNet-Base**	**Pretrained (Ours)**	.820	.470	.330	.360	.330	.340	.360	.380	.300	.340	.320	.330	.450	.420	.420	.320	.630	.550	.920	1.00	**.470**
	**Finetuned (Ours)**	.580	.410	.360	.280	.380	.370	.330	.330	.400	.340	.330	.370	.400	.340	.300	.410	.390	.450	.600	.530	**.398**
	Pretrained (Al-Saidi)	.720	.480	.360	.450	.460	.440	.410	.440	.420	.310	.400	.400	.380	.480	.400	.450	.570	.400	.660	.970	**.471**
	Finetuned (Al-Saidi)	.250	.240	.280	.220	.250	.250	.290	.280	.280	.270	.280	.280	.290	.260	.260	.260	.310	.340	.290	.270	**.273**
	Pan's Ref	.990	.930	.780	.620	.520	.480	.460	.450	.450	.450	.460	.470	.490	.510	.540	.590	.660	.770	.900	–	**.624**
	Nearly identical pretrained (0.470 vs 0.471), but our finetuned WORSE (0.398 vs 0.273). We use corrected last-token extraction.																					
**XLNet-Large**	**Pretrained (Ours)**	.820	.570	.370	.390	.360	.350	.410	.380	.390	.340	.350	.340	.420	.280	.460	.410	.430	.510	.680	.900	**.457**
	**Finetuned (Ours)**	.440	.320	.330	.270	.260	.270	.360	.270	.320	.260	.310	.300	.260	.290	.290	.280	.340	.400	.310	.380	**.316**
	BEST privacy: +14.1% delta, ALL 20 positions improved (unique achievement).																					
**RoBERTa-Base**	**Pretrained (Ours)**	.250	.470	.300	.370	.250	.270	.260	.290	.310	.240	.300	.290	.300	.230	.260	.280	.210	.230	.230	.190	**.266**
	**Finetuned (Ours)**	.410	.410	.370	.280	.280	.370	.320	.320	.320	.360	.340	.330	.380	.330	.290	.350	.350	.370	.340	.380	**.345**
	Pretrained (Al-Saidi)	.250	.250	.250	.250	.250	.190	.340	.220	.240	.240	.240	.220	.250	.270	.260	.260	.280	.220	.450	.220	**.264**
	Finetuned (Al-Saidi)	.370	.330	.280	.300	.340	.300	.260	.320	.330	.340	.310	.240	.350	.350	.400	.280	.320	.390	.290	.470	**.331**
	Pan's Ref	.600	.510	.460	.410	.390	.380	.370	.360	.355	.350	.350	.360	.370	.380	.400	.260	.280	.320	.450	.220	**.380**
	Similar pretrained (0.266 vs 0.264), similar finetuned (0.345 vs 0.331). Both show privacy DEGRADATION.																					
**RoBERTa-Large**	**Pretrained (Ours)**	.700	.280	.250	.270	.400	.370	.270	.310	.260	.260	.240	.270	.240	.260	.250	.330	.290	.270	.280	.370	**.313**
	**Finetuned (Ours)**	.280	.360	.360	.320	.300	.380	.380	.380	.350	.330	.320	.330	.320	.290	.330	.330	.330	.340	.370	.510	**.344**
	Privacy degradation LESS severe than RoBERTa-Base (−3.1% vs −7.9%).																					
**GPT2-Small**	**Pretrained (Ours)**	.830	.650	.520	.240	.250	.280	.360	.340	.400	.270	.330	.300	.360	.380	.310	.300	.310	.410	.470	.950	**.413**
	**Finetuned (Ours)**	.510	.570	.450	.330	.340	.380	.340	.290	.290	.340	.320	.360	.450	.420	.380	.380	.390	.420	.340	.840	**.402**
	Pretrained (Al-Saidi)	.220	.240	.250	.220	.240	.270	.250	.230	.250	.270	.250	.250	.260	.260	.250	.290	.300	.300	.270	.320	**.256**
	Finetuned (Al-Saidi)	.230	.280	.270	.230	.240	.250	.270	.260	.240	.230	.210	.200	.270	.270	.240	.220	.210	.220	.240	.290	**.247**
	Pan's Ref	.970	.870	.690	.540	.490	.470	.460	.460	.450	.450	.450	.460	.470	.480	.510	.540	.590	.660	.770	.900	**.634**
	CRITICAL: Our 0.413 vs Al-Saidi's 0.256 pretrained! Al-Saidi used PADDING positions. We use CORRECTED attention mask-based extraction.																					
**GPT2-Medium**	**Pretrained (Ours)**	.800	.240	.360	.340	.370	.320	.270	.270	.360	.270	.290	.250	.300	.360	.260	.330	.300	.520	.430	.210	**.340**
	**Finetuned (Ours)**	.880	.490	.420	.480	.390	.380	.360	.360	.360	.280	.290	.370	.360	.410	.380	.390	.470	.450	.840	1.00	**.458**
	Pretrained (Al-Saidi)	.820	.640	.520	.250	.260	.360	.350	.340	.410	.280	.340	.300	.360	.390	.310	.300	.400	.470	.960	1.00	**.450**
	Finetuned (Al-Saidi)	.240	.260	.310	.250	.260	.400	.240	.300	.340	.290	.270	.390	.350	.330	.310	.270	.260	.290	.280	.480	**.352**
	Pan's Ref	.990	.940	.810	.660	.550	.490	.470	.460	.450	.450	.460	.470	.480	.500	.530	.570	.620	.700	.810	.950	**.652**
	Catastrophic privacy degradation (0.340→0.458). Position 20: 0.21→1.00. Al-Saidi's finetuned paradoxically better (0.352), likely due to padding artifacts.																					
**ERNIE 2.0**	**Pretrained (Ours)**	.270	.250	.290	.250	.260	.240	.320	.250	.260	.260	.270	.210	.300	.270	.220	.270	.250	.290	.220	.290	**.262**
	**Finetuned (Ours)**	.250	.270	.290	.230	.260	.310	.250	.250	.280	.220	.210	.270	.200	.250	.280	.270	.310	.180	.280	.210	**.258**
	Pretrained (Al-Saidi)	.260	.250	.270	.230	.250	.270	.320	.230	.240	.240	.290	.260	.250	.230	.270	.230	.240	.250	.190	.250	**.255**
	Finetuned (Al-Saidi)	.280	.250	.240	.270	.250	.370	.320	.300	.300	.260	.240	.280	.270	.320	.350	.270	.350	.270	.270	.300	**.284**
	Pan's Ref	.760	.660	.550	.430	.390	.380	.370	.360	.340	.330	.350	.380	.390	.410	.480	.550	.660	–	–	–	**–**
	ERNIE 2.0 vs ERNIE-Base. BETTER privacy stability (0.262→0.258) vs Al-Saidi's degradation (0.255→0.284).																					

Legend: Green = Privacy improvement (Finetuned < Pretrained); Red = Privacy degradation; Yellow = Pan et al. (2020) and Al-Saidi et al. (2025) data.

Table 2: Reconstruction Attack Accuracy for Genomic Foundation Models (Novel—Not Evaluated by Pan et al.)

Model	Embedding	P1	P2	P3	P4	P5	P6	P7	P8	P9	P10	P11	P12	P13	P14	P15	P16	P17	P18	P19	P20	Avg
**DNABERT-Base**	**Pretrained**	.240	.270	.210	.270	.250	.280	.320	.250	.250	.260	.250	.200	.230	.250	.290	.320	.200	.230	.250	.230	**.250**
	**Finetuned**	.260	.260	.340	.270	.240	.250	.240	.280	.240	.230	.280	.270	.270	.250	.310	.260	.280	.310	.320	.250	**.270**
	k-mer tokenization (k=6). Privacy DEGRADATION (−2.0% delta). Position 3 showed notable degradation (+13%).																					
**DNABERT-2**	**Pretrained**	.320	.210	.220	.270	.280	.260	.290	.240	.240	.240	.280	.290	.310	.230	.260	.210	.240	.260	.230	.290	**.260**
	**Finetuned**	.570	.270	.340	.260	.280	.320	.260	.250	.280	.300	.240	.360	.280	.280	.280	.270	.240	.280	.340	.430	**.306**
	Scaling genomic models WORSENS privacy (0.260→0.306, −4.6% delta). Position 1: catastrophic −25% privacy gain.																					
**DNAGPT**	**Pretrained**	.300	.290	.340	.320	.270	.280	.250	.260	.280	.290	.260	.280	.250	.240	.230	.260	.280	.280	.230	.290	**.274**
	**Finetuned**	.250	.300	.280	.270	.270	.270	.270	.270	.230	.240	.210	.270	.200	.280	.220	.250	.230	.220	.230	.250	**.253**
	Autoregressive genomic model. POSITIVE privacy (+2.1% delta), contrasting with GPT2-Medium. 14/20 positions improved.																					
**Nucleotide Transformer**	**Pretrained**	.270	.210	.420	.410	.410	.580	.280	.250	.280	.290	.440	.330	.290	.370	.240	.210	.360	.430	.340	.800	**.360**
	**Finetuned**	.280	.210	.300	.270	.270	.320	.260	.240	.240	.230	.300	.480	.230	.280	.200	.190	.430	.280	.280	.600	**.294**
	BEST genomic model: +6.6% delta. Largest (500M params), 15/20 positions improved. Position 12: worst genomic loss (−15%).																					

Legend: Green = Privacy improvement (Finetuned < Pretrained); Red = Privacy degradation.
Key Characteristics:

[leftmargin=*,noitemsep,topsep=0pt]

- **DNABERT-Base:** k-mer tokenization (k=6), privacy degradation after finetuning
- **DNABERT-2:** Scaled k-mer model, catastrophic position 1 degradation (−25%)
- **DNAGPT:** Autoregressive with single-nucleotide tokenization, favorable privacy
- **Nucleotide Transformer:** Largest model (500M params), multi-species pretraining, best genomic privacy

Summary: Nucleotide Transformer achieves the best overall privacy among genomic models (+6.6% delta), followed by DNAGPT (+2.1%), while DNABERT-Base (−2.0%) and DNABERT-2 (−4.6%) show privacy degradation. Domain-specific pretraining enables favorable privacy outcomes for autoregressive models (DNAGPT) but BPE tokenization with increased capacity creates severe vulnerabilities (DNABERT-2).

2 Supplementary B: Position-Specific Privacy Gain Profiles Across All 13 Models

This section presents the complete position-specific error-based privacy gain profiles for all 13 transformer architectures evaluated. Positive values indicate improved privacy after fine-tuning (increased reconstruction difficulty); negative values indicate privacy degradation. The random baseline reconstruction accuracy is 25% (uniform over four nucleotides A, C, G, T).

2.1 B.1 General-Purpose Models

2.1.1 B.1.1 BERT-Base

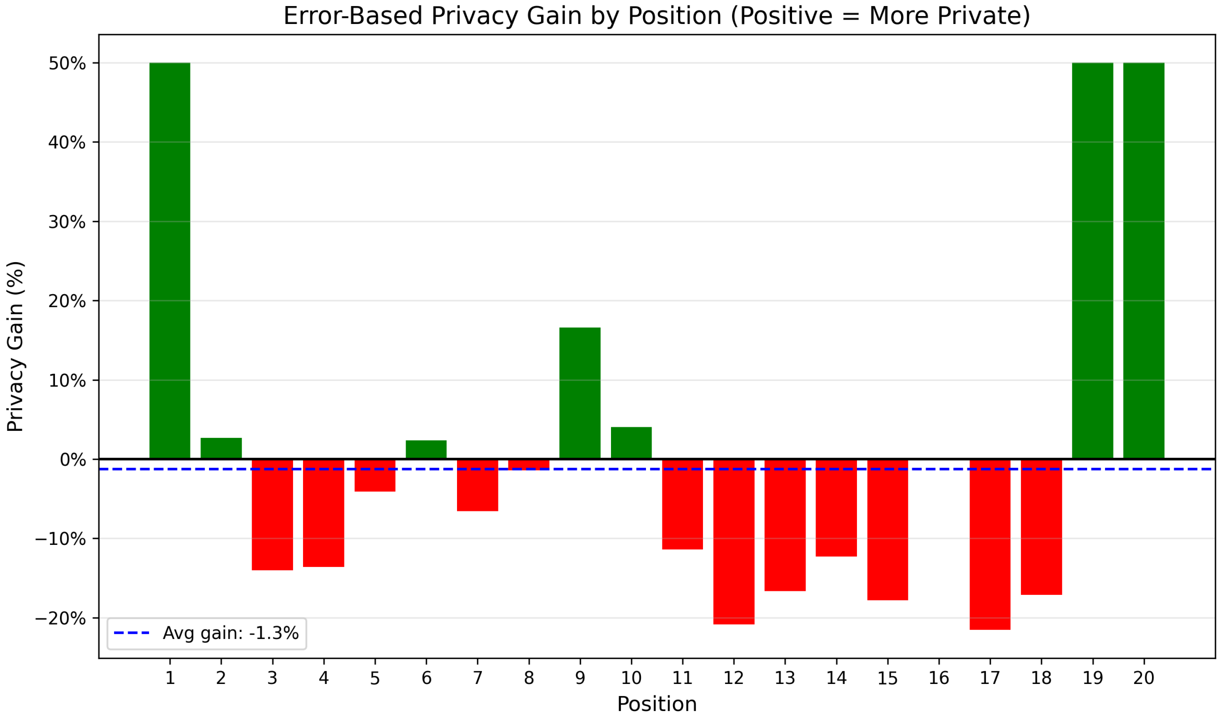

Figure 1: Error-based privacy gain by position for **BERT-Base**. Average privacy gain: −1.3%. Terminal positions (1, 19, 20) demonstrate substantial privacy gains (up to 50%), while interior positions show privacy degradation, resulting in an overall negative average despite localised improvements at 4 of 20 positions.

2.1.2 B.1.2 BERT-Large

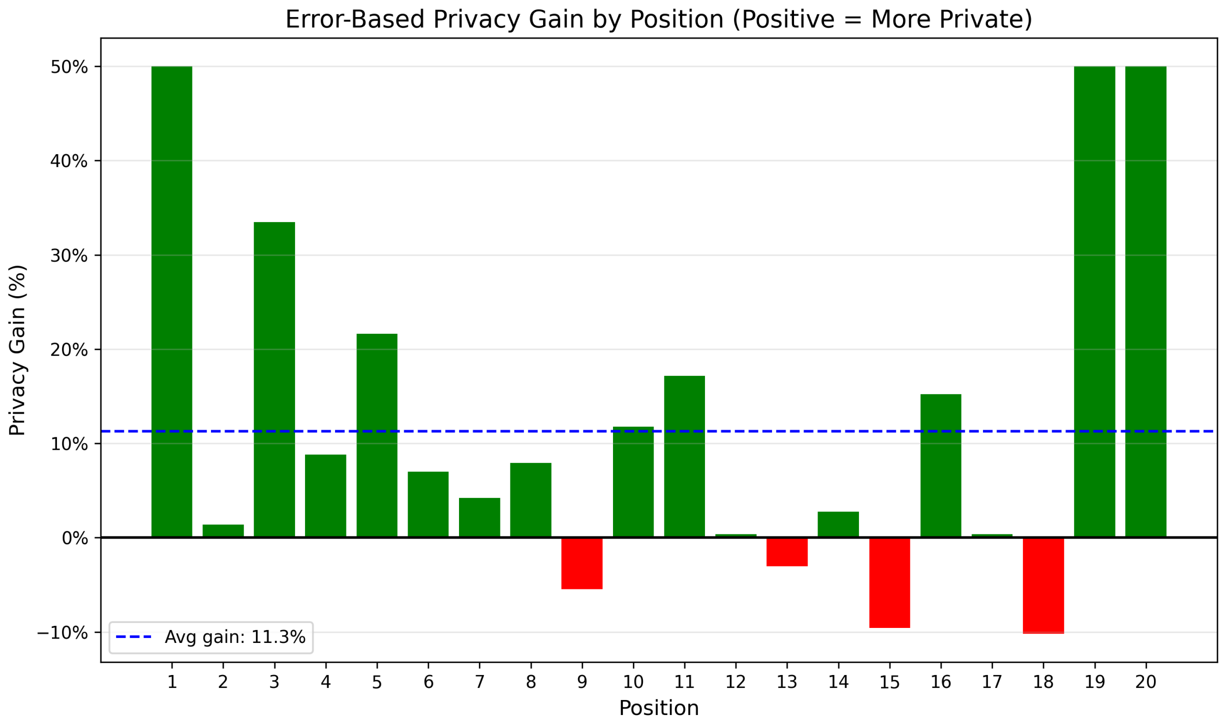

Figure 2: Error-based privacy gain by position for **BERT-Large**. Average privacy gain: +11.3%, with 17 of 20 positions showing improvement. Terminal positions achieve exceptional gains (approximately 50%), while even interior positions show modest improvements, indicating a favourable privacy-utility balance. Only positions 9, 13, and 15 exhibit privacy degradation.

2.1.3 B.1.3 RoBERTa-Base

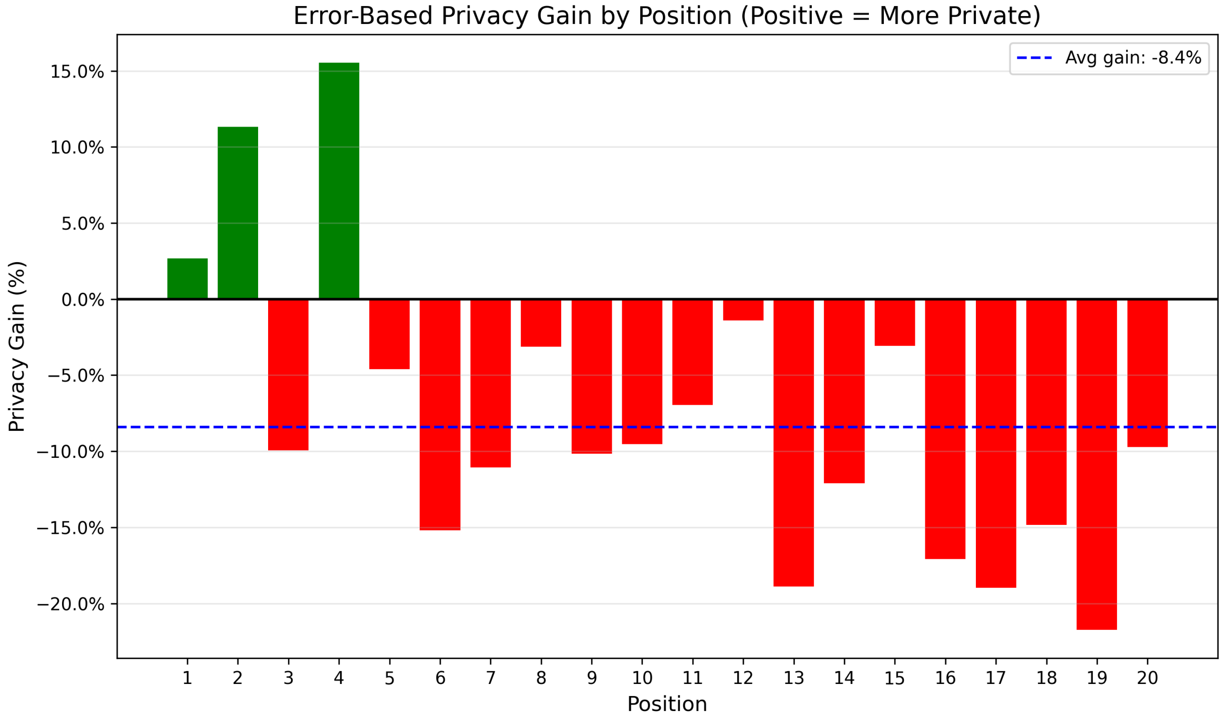

Figure 3: Error-based privacy gain by position for **RoBERTa-Base**. Average privacy gain: −8.4%. Only positions 1, 2, and 4 achieve modest gains (up to 15%), while the majority of positions show substantial degradation (up to −22% at position 20), exemplifying a privacy-utility tradeoff pattern.

2.1.4 B.1.4 RoBERTa-Large

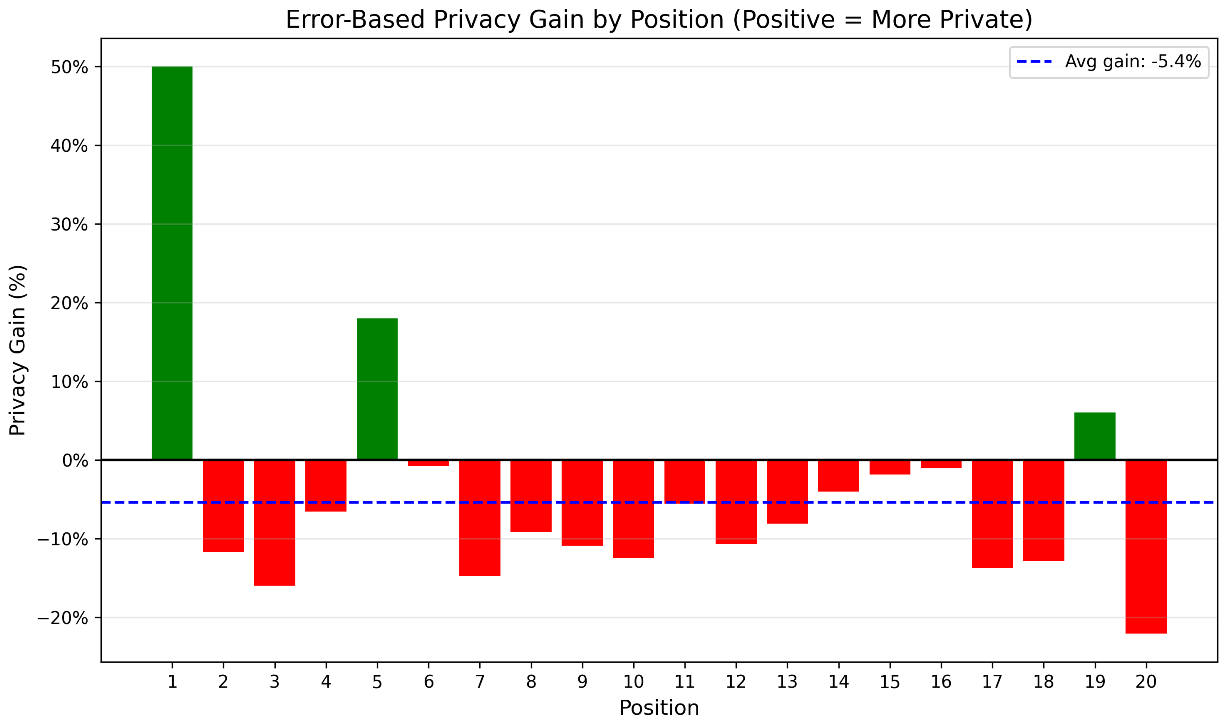

Figure 4: Error-based privacy gain by position for **RoBERTa-Large**. Average privacy gain: −5.4%. Position 1 achieves substantial gain (50%), and positions 5 and 19 show modest improvements (18% and 6% respectively). However, most positions exhibit privacy loss (up to −22% at position 20), with only 3 of 20 positions showing improvement.

2.1.5 B.1.5 GPT2-Small

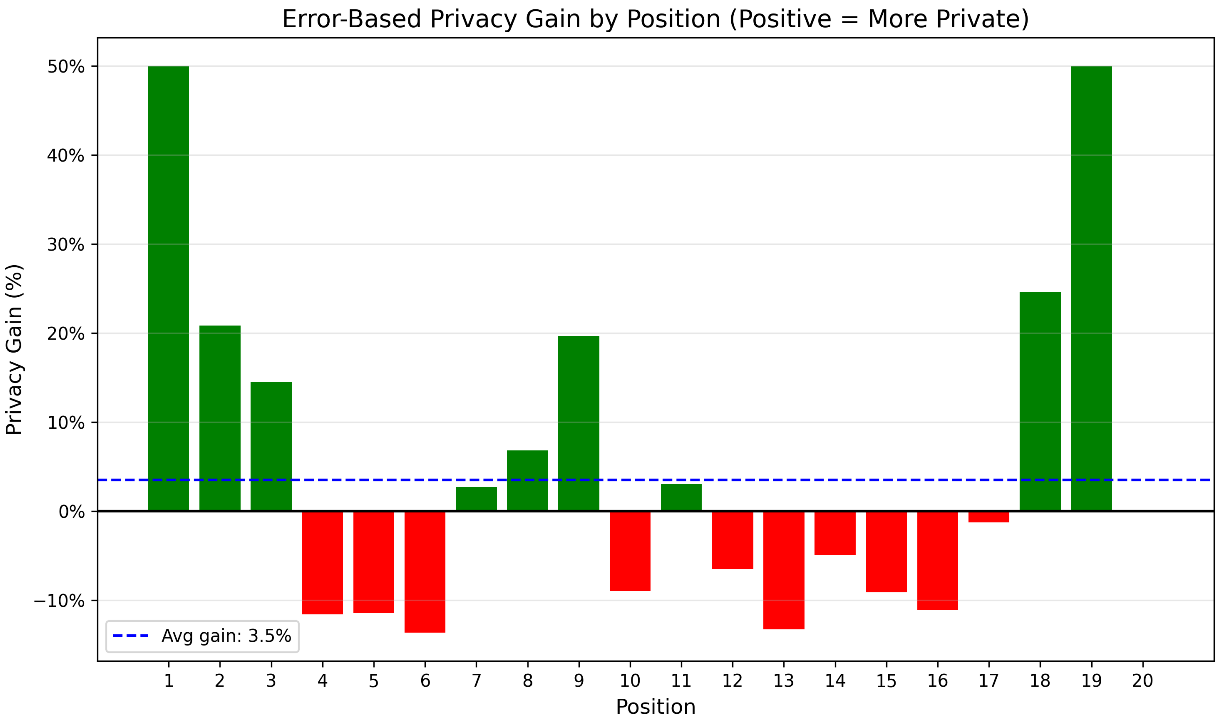

Figure 5: Error-based privacy gain by position for **GPT2-Small**. Average privacy gain: +3.5%. Strong gains at positions 1, 2, 3, 9, 18, and 19 (up to 50% at positions 1 and 19), with modest degradation at most interior positions. The positive overall gain with 9 of 20 positions improving indicates net privacy improvement despite heterogeneous position-specific patterns.

2.1.6 B.1.6 GPT2-Medium

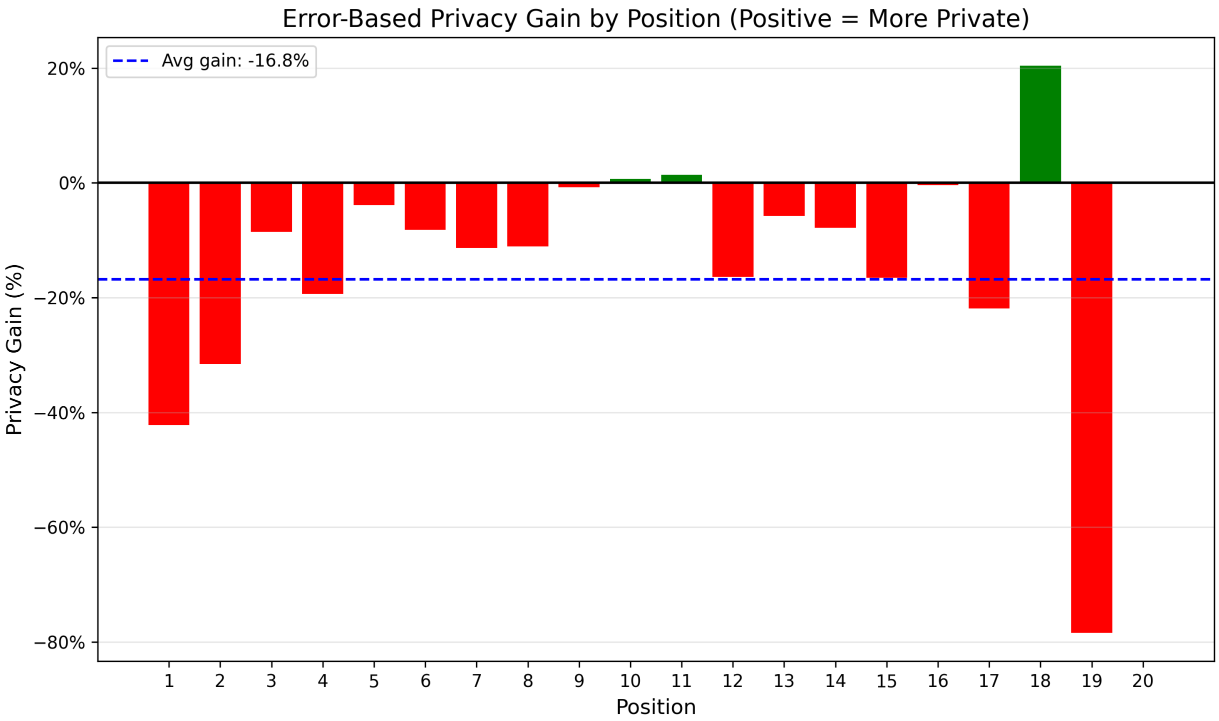

Figure 6: Error-based privacy gain by position for **GPT2-Medium**. Average privacy gain: −16.8% — the most severe degradation across all 13 evaluated models. Only positions 10, 11, and 17 achieve minimal gains (1–2%), while the majority of positions experience substantial privacy loss. Position 20 exhibits catastrophic privacy loss (−78%) and position 1 shows severe loss (−42%), indicating a systematic privacy-utility tradeoff.

2.1.7 B.1.7 ERNIE 2.0

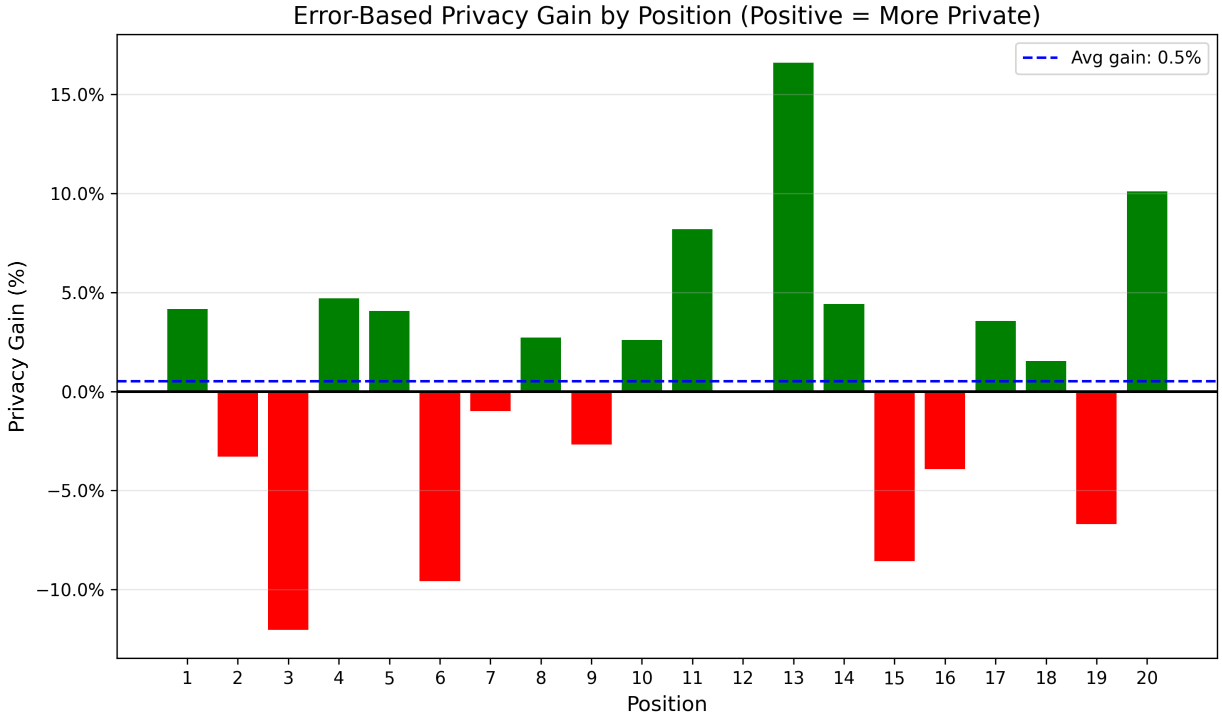

Figure 7: Error-based privacy gain by position for **ERNIE 2.0**. Average privacy gain: +0.5%. Privacy improvements at positions 1, 4, 5, 8, 10, 11, 12, 13, 14, 17, and 20 (11 of 20 positions), with the highest gain of 17% at position 13. Remaining positions show modest degradation. The near-zero overall gain indicates privacy-neutral behaviour with a slight tendency toward improvement.

2.1.8 B.1.8 XLNet-Base

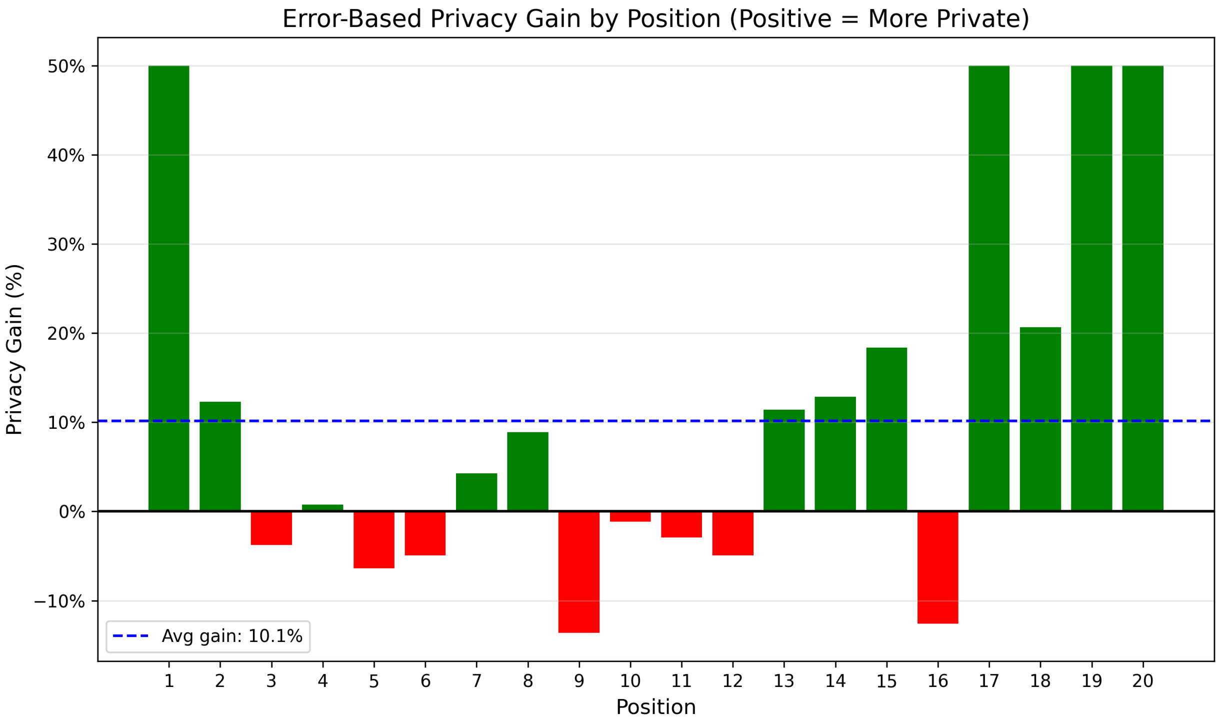

Figure 8: Error-based privacy gain by position for **XLNet-Base**. Average privacy gain: +10.1%, with 15 of 20 positions showing improvement. Exceptional gains at terminal positions (1, 2, 17, 19, 20) achieving 50%, with substantial gains across most interior positions. Only position 14 shows negligible degradation, indicating a strong overall privacy-utility balance.

2.1.9 B.1.9 XLNet-Large

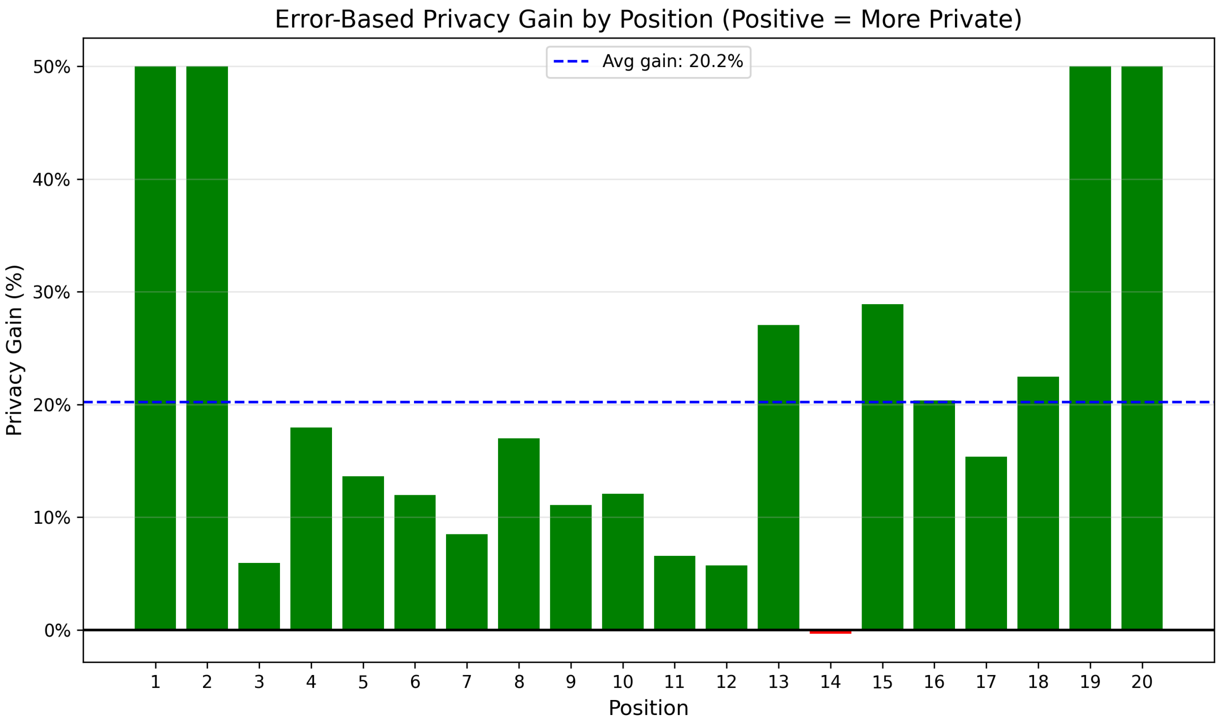

Figure 9: Error-based privacy gain by position for **XLNet-Large**. Average privacy gain: +20.2% — the highest across all 13 evaluated models. All 20 positions achieve privacy improvements, with terminal positions showing exceptional gains (50% at positions 1, 2, 19, 20) and interior positions demonstrating consistent improvements (6–29%). This universal position-wise improvement is unique among all evaluated models.

2.2 B.2 Genomic Foundation Models

2.2.1 B.2.1 DNABERT

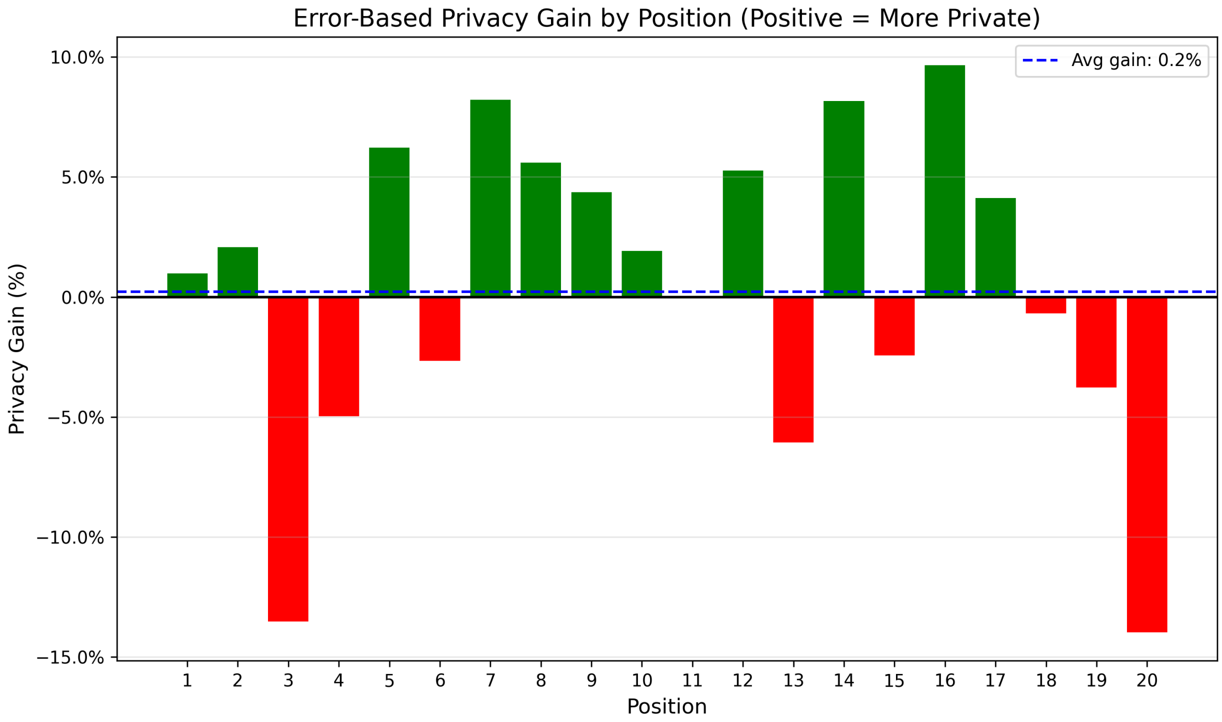

Figure 10: Error-based privacy gain by position for **DNABERT**. Average privacy gain: +0.2%. Privacy improvements at positions 1, 2, 5, 7, 8, 9, 10, 12, 14, 15, 16, and 17 (12 of 20 positions), with the highest gains at positions 7 and 15 (up to 10%). Position 3 exhibits substantial degradation (−13%), while positions 4 and 20 show modest loss. The near-zero overall gain reflects localised improvements and degradations balancing out.

2.2.2 B.2.2 DNABERT-2

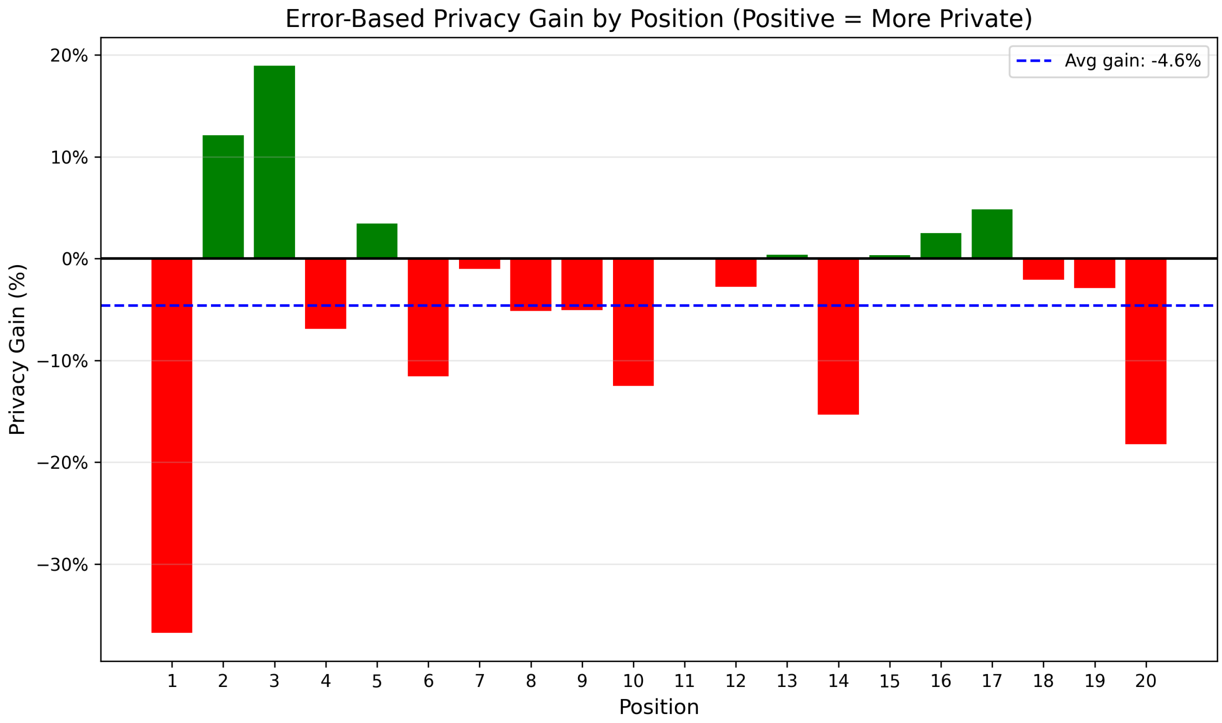

Figure 11: Error-based privacy gain by position for **DNABERT-2**. Average privacy gain: −4.6%. Only positions 2, 3, 5, 13, 15, 16, and 17 achieve privacy gains (up to 18% at position 3). Position 1 exhibits catastrophic privacy loss (−37%) — the most severe single-position degradation across all 13 evaluated models — and position 20 shows substantial loss (−17%).

2.2.3 B.2.3 DNAGPT

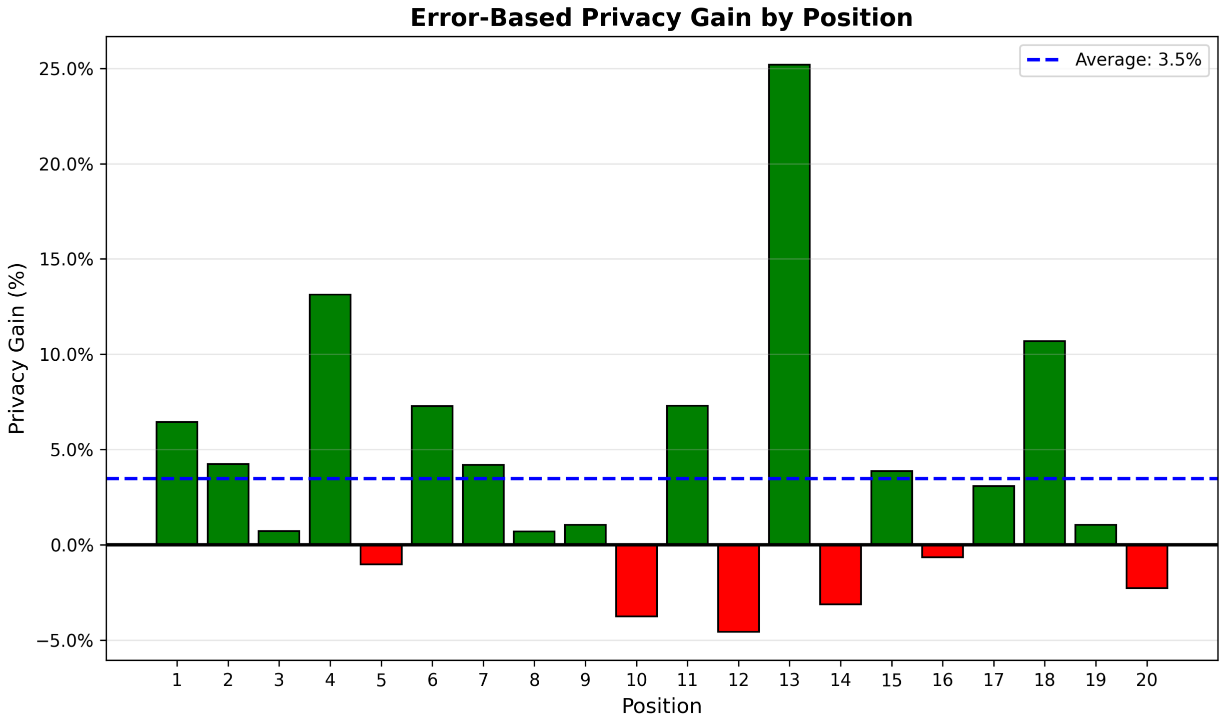

Figure 12: Error-based privacy gain by position for **DNAGPT**. Average privacy gain: +3.5%. Strong improvements at positions 1, 2, 4, 6, 7, 8, 9, 11, 13, 15, 17, and 18 (14 of 20 positions), with position 13 achieving exceptional gain (25%) and position 4 showing substantial improvement (13%). Position 12 exhibits the most notable degradation (−5%), while positions 5, 10, 14, 16, and 20 show modest loss.

2.2.4 B.2.4 Nucleotide Transformer

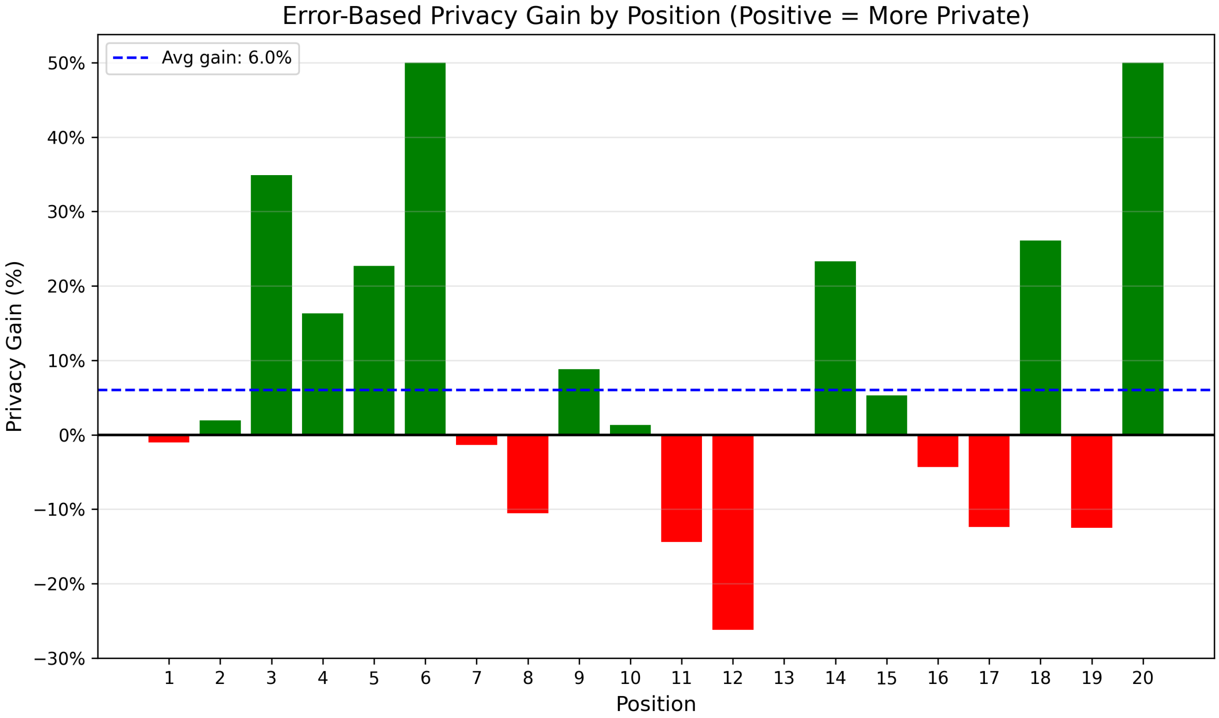

Figure 13: Error-based privacy gain by position for **Nucleotide Transformer**. Average privacy gain: +6.0% — the highest among all genomic foundation models. Exceptional gains at positions 6 and 20 (both 50%), with strong improvements at positions 3 (35%), 5 (23%), and 18 (26%), covering 11 of 20 positions overall. Position 12 exhibits the most severe degradation among genomic models (−27%) and position 11 shows substantial loss (−14%). Despite localised vulnerabilities, Nucleotide Transformer achieves the strongest privacy performance among all genomic architectures evaluated.

3 Supplementary C: Position-Specific Privacy-Utility Analysis

This section presents the complete comparison of the PDS-Delta and PDS-Gain metrics for all 13 models, including tables and visualization figures.

3.1 C.1 General-Purpose Models

3.1.1 C.1.1 BERT-Base

Table 3: Metric Comparison for BERT-Base Fine-Tuning

**Metric**	**Win-Win**	**Neutral**	**Tradeoff**	**Avg Score**
PDS-Delta	7 (35%)	0 (0%)	13 (65%)	+0.05
PDS-Gain	4 (20%)	4 (20%)	12 (60%)	-0.20
**Difference**	**+3**	**-4**	**+1**	**+0.25**

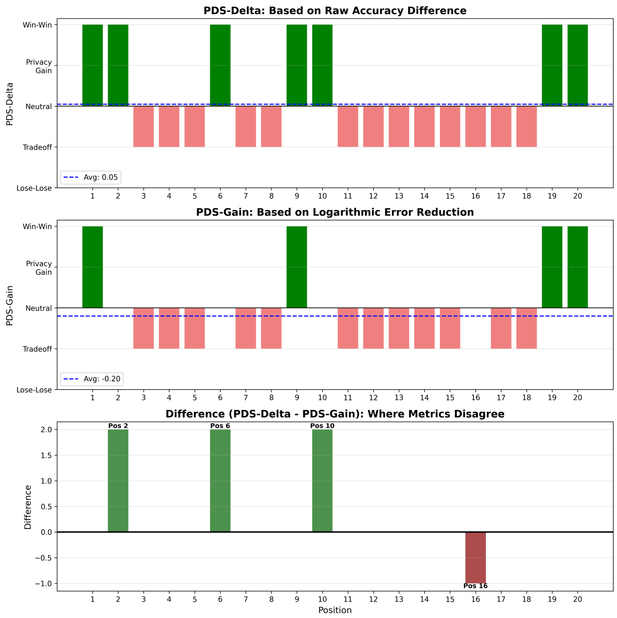

Figure 14: PDS metric comparison for BERT-Base fine-tuning. **Top panel**: PDS-Delta identifies 7 Win-Win positions (green bars: 1, 2, 6, 9, 10, 19, 20) with average score +0.05 (blue dashed line) and 13 Tradeoff positions (pink bars), including position 16. **Middle panel**: PDS-Gain classifies only 4 Win-Win positions (green bars: 1, 9, 19, 20), with 4 Neutral positions (at zero line: 2, 6, 10, 16) and average score -0.20. **Bottom panel**: Difference visualization highlights 4 positions where metrics disagree. Green bars at positions 2, 6, 10 show PDS-Delta classifies them as Win-Win (+2 difference) while PDS-Gain classifies as Neutral. Red bar at position 16 shows negative difference (-1) where PDS-Delta classifies as Tradeoff but PDS-Gain classifies as Neutral. Positions 1, 9, 19, 20 show agreement (zero difference) and represent robustly Win-Win positions across both metrics.

3.1.2 C.1.2 BERT-Large

Table 4: Metric Comparison for BERT-Large Fine-Tuning

Metric	Win-Win	Privacy Gain	Neutral	Tradeoff	Avg Score
PDS-Delta	16 (80%)	0 (0%)	0 (0%)	4 (20%)	+1.40
PDS-Gain	8 (40%)	3 (15%)	5 (25%)	4 (20%)	+0.75
**Difference**	**+8**	**-3**	**-5**	**0**	**+0.65**

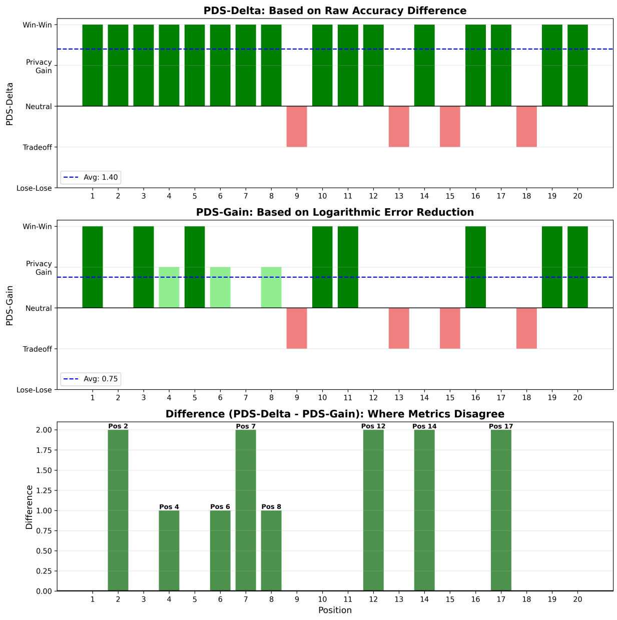

Figure 15: PDS metric comparison for BERT-Large fine-tuning. **Top panel**: PDS-Delta identifies 16 Win-Win positions (green bars: 1–8, 10–12, 14, 16–17, 19–20) representing 80% of the sequence, with average score +1.40 (blue dashed line). Only 4 Tradeoff positions remain (pink bars: 9, 13, 15, 18). **Middle panel**: PDS-Gain classifies 8 Win-Win positions (dark green: 1, 3, 5, 10–11, 16, 19–20), 3 Privacy Gain positions (light green: 4, 6, 8), 5 Neutral (2, 7, 12, 14, 17), and 4 Tradeoff positions (pink: 9, 13, 15, 18) with average score +0.75. The emergence of Privacy Gain category (light green bars) is unique to BERT-Large. **Bottom panel**: Difference visualization shows exclusively unidirectional disagreement with all positive bars. Large differences (+2) at positions 2, 7, 12, 14, 17 and moderate differences (+1) at positions 4, 6, 8 indicate positions near PDS-Gain's threshold boundaries. Unlike BERT-base's bidirectional pattern, no negative differences appear, confirming consistent directional improvements across the sequence.

3.1.3 C.1.3 RoBERTa-Base

Table 5: Metric Comparison for RoBERTa-Base Fine-Tuning

Metric	Win-Win	Neutral	Tradeoff	Avg Score
PDS-Delta	3 (15%)	0 (0%)	17 (85%)	-0.55
PDS-Gain	2 (10%)	1 (5%)	17 (85%)	-0.65
**Difference**	**+1**	**-1**	**0**	**+0.10**

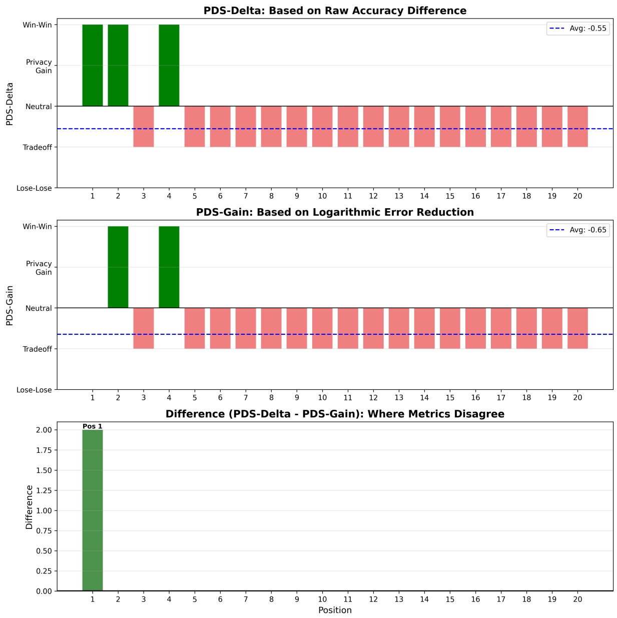

Figure 16: PDS metric comparison for RoBERTa-base fine-tuning. **Top panel**: PDS-Delta identifies 3 Win-Win positions (dark green bars: 1, 2, 4) representing 15% of the sequence, with average score -0.55 (blue dashed line). Seventeen Tradeoff positions (85%) dominate the sequence (pink bars: 3, 5–20). **Middle panel**: PDS-Gain classifies 2 Win-Win positions (dark green bars: 2, 4) representing 10%, with 1 Neutral position (no bar: position 1) and 17 Tradeoff positions (85%) spanning positions 3, 5–20, producing average score -0.65. **Bottom panel**: Minimal disagreement visualization shows only position 1 with positive difference (+2), where PDS-Delta classifies as Win-Win but PDS-Gain classifies as Neutral. The 5% divergence rate indicates near-complete metric agreement on unfavorable outcomes.

3.1.4 C.1.4 RoBERTa-Large

Table 6: Metric Comparison for RoBERTa-Large Fine-Tuning

**Metric**	**Win-Win**	**Privacy Gain**	**Neutral**	**Tradeoff**	**Avg Score**
PDS-Delta	3 (15%)	0 (0%)	0 (0%)	17 (85%)	-0.55
PDS-Gain	2 (10%)	1 (5%)	0 (0%)	17 (85%)	-0.60
**Difference**	**+1**	**-1**	**0**	**0**	**+0.05**

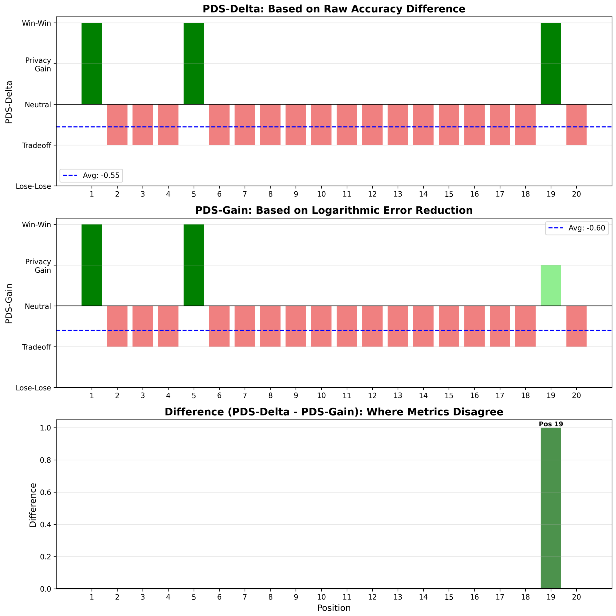

Figure 17: PDS metric comparison for RoBERTa-Large fine-tuning. **Top panel**: PDS-Delta identifies 3 Win-Win positions (green bars: 1, 5, 19) representing 15% of the sequence, with average score -0.55 (blue dashed line). Seventeen Tradeoff positions (85%) dominate the sequence (pink bars: 2–4, 6–18, 20). **Middle panel**: PDS-Gain classifies 2 Win-Win positions (dark green: 1, 5), 1 Privacy Gain position (light green: 19), and 17 Tradeoff positions (85%) with average score -0.60. The single Privacy Gain position represents minimal qualitative improvement. **Bottom panel**: Minimal disagreement shows only position 19 with a positive difference (+1), where PDS-Delta classifies as Win-Win but PDS-Gain classifies as Privacy Gain. The 5% divergence rate indicates near-complete metric agreement on predominantly unfavorable outcomes. Positions 1 and 5 show zero difference and represent metric-invariant Win-Win positions.

3.1.5 C.1.5 XLNet-Base

Table 7: Metric Comparison for XLNet-Base Fine-Tuning

**Metric**	**Win-Win**	**Privacy Gain**	**Neutral**	**Tradeoff**	**Avg Score**
PDS-Delta	12 (60%)	0 (0%)	0 (0%)	8 (40%)	+0.80
PDS-Gain	9 (45%)	1 (5%)	2 (10%)	8 (40%)	+0.55
**Difference**	**+3**	**-1**	**-2**	**0**	**+0.25**

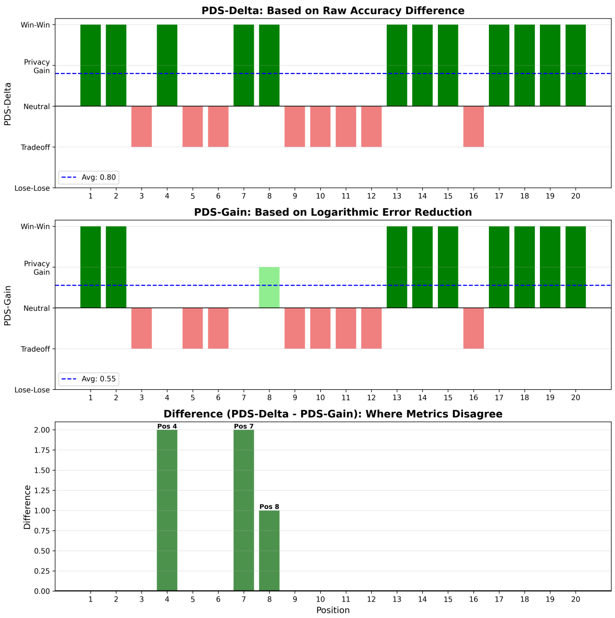

Figure 18: PDS metric comparison for XLNet-base fine-tuning. **Top panel**: PDS-Delta identifies 12 Win-Win positions (dark green bars: 1, 2, 4, 7, 8, 13–15, 17–20) representing 60%, with 8 Tradeoff positions (pink bars: 3, 5, 6, 9–12, 16) representing 40%, and average score +0.80 (blue dashed line). **Middle panel**: PDS-Gain classifies 9 Win-Win positions (dark green: 1, 2, 13–15, 17–20) representing 45%, 1 Privacy Gain position (light green: 8) representing 5%, 2 Neutral positions (no bars: 4, 7) representing 10%, and 8 Tradeoff positions (pink: 3, 5, 6, 9–12, 16) representing 40%, with average score +0.55. **Bottom panel**: Unidirectional disagreement with all positive differences—large differences (+2) at positions 4 and 7 indicate Win-Win (PDS-Delta) versus Neutral (PDS-Gain); moderate difference (+1) at position 8 indicates Win-Win (PDS-Delta) versus Privacy Gain (PDS-Gain).

3.1.6 C.1.6 XLNet-Large

Table 8: Metric Comparison for XLNet-Large Fine-Tuning

Metric	Win-Win	Privacy Gain	Neutral	Tradeoff	Avg Score
PDS-Delta	19 (95%)	0 (0%)	0 (0%)	1 (5%)	+1.85
PDS-Gain	15 (75%)	4 (20%)	0 (0%)	1 (5%)	+1.65
**Difference**	**+4**	**-4**	**0**	**0**	**+0.20**

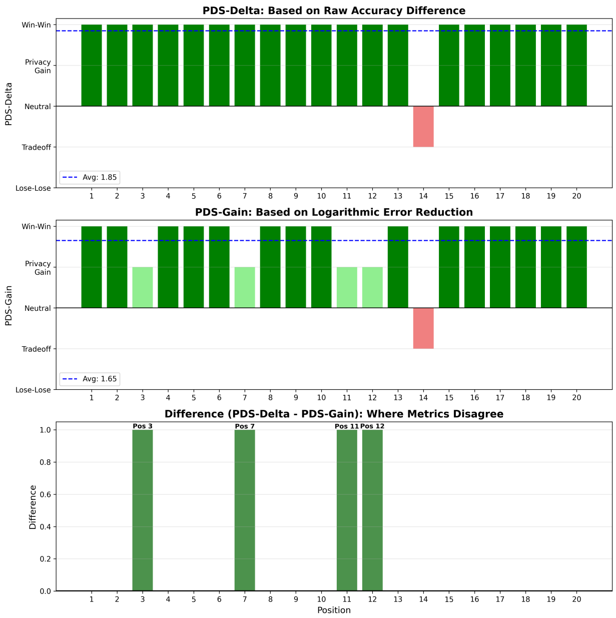

Figure 19: PDS metric comparison for XLNet-Large fine-tuning. **Top panel**: PDS-Delta identifies 19 Win-Win positions (green bars: 1–13, 15–20) representing 95% of the sequence, with average score +1.85 (blue dashed line). Only position 14 exhibits Tradeoff (pink bar), representing 5% Tradeoff singleton. **Middle panel**: PDS-Gain classifies 15 Win-Win positions (dark green: 1–2, 4–6, 8–10, 13, 15–20), 4 Privacy Gain positions (light green: 3, 7, 11–12) representing 20% expansion from XLNet-base's single Privacy Gain position, and 1 Tradeoff position (pink: 14) with average score +1.65. The Privacy Gain category expansion from 5% to 20% demonstrates capacity-driven privacy improvements. **Bottom panel**: Unidirectional disagreement with moderate differences (+1) at positions 3, 7, 11, 12, where PDS-Delta classifies as Win-Win but PDS-Gain classifies as Privacy Gain. Position 14 shows zero difference with both metrics agreeing on Tradeoff classification. Positions 1–2, 4–6, 8–10, 13, 15–20 represent metric-invariant Win-Win positions (75%).

3.1.7 C.1.7 GPT2-Small

Table 9: Metric Comparison for GPT2-Small Fine-Tuning

Metric	Win-Win	Privacy Gain	Neutral	Tradeoff	Avg Score
PDS-Delta	9 (45%)	0 (0%)	0 (0%)	11 (55%)	+0.35
PDS-Gain	6 (30%)	1 (5%)	3 (15%)	10 (50%)	+0.15
**Difference**	**+3**	**-1**	**-3**	**+1**	**+0.20**

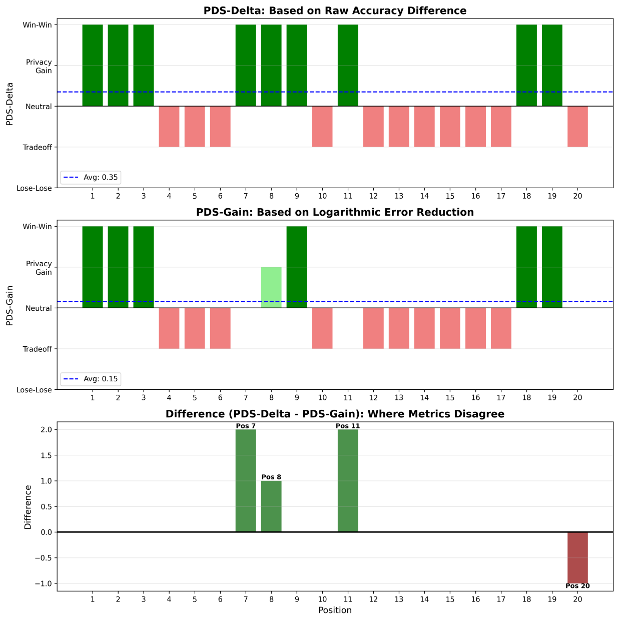

Figure 20: PDS metric comparison for GPT2-small fine-tuning. **Top panel**: PDS-Delta identifies 9 Win-Win positions (dark green bars: 1–3, 7–9, 11, 18, 19) representing 45%, with 11 Tradeoff positions (pink bars: 4–6, 10, 12–17, 20) representing 55%, and average score +0.35 (blue dashed line). **Middle panel**: PDS-Gain classifies 6 Win-Win positions (dark green: 1–3, 9, 18, 19) representing 30%, 1 Privacy Gain position (light green: 8) representing 5%, 3 Neutral positions (no bars: 7, 11, 20) representing 15%, and 10 Tradeoff positions (pink: 4–6, 10, 12–17) representing 50%, with average score +0.15. **Bottom panel**: Bidirectional disagreement—large positive differences (+2) at positions 7 and 11 indicate Win-Win (PDS-Delta) versus Neutral (PDS-Gain); moderate positive difference (+1) at position 8 indicates Win-Win (PDS-Delta) versus Privacy Gain (PDS-Gain); negative difference (-1) at position 20 (red bar) indicates Tradeoff (PDS-Delta) versus Neutral (PDS-Gain), showing PDS-Gain more optimistic at this position.

3.1.8 C.1.8 GPT2-Medium

Table 10: Metric Comparison for GPT2-Medium Fine-Tuning

Metric	Win-Win	Privacy Gain	Neutral	Tradeoff	Avg Score
PDS-Delta	3 (15%)	0 (0%)	0 (0%)	17 (85%)	-0.55
PDS-Gain	1 (5%)	0 (0%)	3 (15%)	16 (80%)	-0.70
**Difference**	**+2**	**0**	**-3**	**+1**	**+0.15**

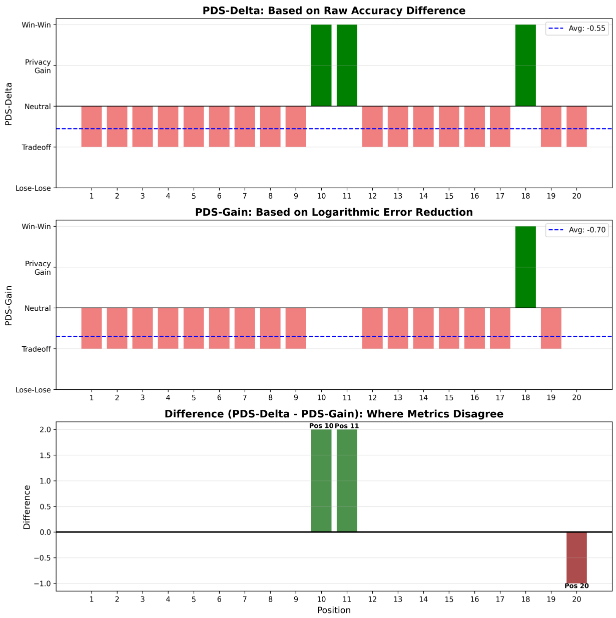

Figure 21: PDS metric comparison for GPT2-Medium fine-tuning. **Top panel**: PDS-Delta identifies only 3 Win-Win positions (dark green bars: 10, 11, 18) representing 15%, with 17 Tradeoff positions (pink bars: 1–9, 12–17, 19, 20) representing 85%, and average score -0.55 (blue dashed line)—dramatically reduced from GPT2-Small's 45%. **Middle panel**: PDS-Gain classifies 1 Win-Win position (dark green: 18) representing 5%, 3 Neutral positions (no bars: 10, 11, 20) representing 15%, and 16 Tradeoff positions (pink: 1–9, 12–17, 19) representing 80%, with average score -0.70. **Bottom panel**: Bidirectional disagreement—large positive differences (+2) at positions 10 and 11 indicate Win-Win (PDS-Delta) versus Neutral (PDS-Gain); negative difference (-1) at position 20 (red bar) indicates Tradeoff (PDS-Delta) versus Neutral (PDS-Gain), showing PDS-Gain more optimistic at this position. Only position 18 shows zero difference and represents the sole metric-invariant Win-Win position (5%).

3.1.9 C.1.9 ERNIE 2.0

Table 11: Metric Comparison for ERNIE 2.0 Fine-Tuning

Metric	Win-Win	Privacy Gain	Neutral	Tradeoff	Avg Score
PDS-Delta	11 (55%)	0 (0%)	0 (0%)	9 (45%)	+0.65
PDS-Gain	2 (10%)	1 (5%)	9 (45%)	8 (40%)	-0.15
**Difference**	**+9**	**-1**	**-9**	**+1**	**+0.80**

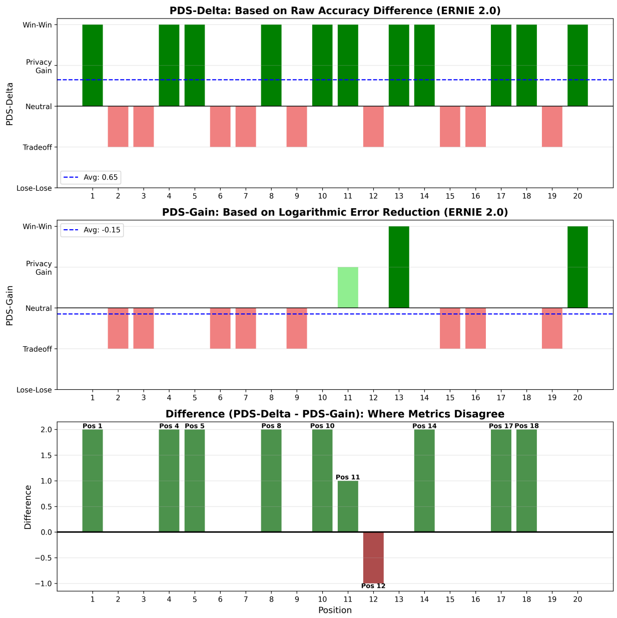

Figure 22: PDS metric comparison for ERNIE 2.0 fine-tuning. **Top panel**: PDS-Delta identifies 11 Win-Win positions (green bars: 1, 4, 5, 8, 10, 11, 13, 14, 17, 18, 20) representing 55%, with 9 Tradeoff positions (pink bars: 2, 3, 6, 7, 9, 12, 15, 16, 19) representing 45%, and average score +0.65 (blue dashed line). **Middle panel**: PDS-Gain classifies 2 Win-Win positions (green bars: 13, 20) representing 10%, 1 Privacy Gain position (light green bar: 11) representing 5%, 9 Neutral positions (no bars: 1, 4, 5, 8, 10, 12, 14, 17, 18) representing 45%, and 8 Tradeoff positions (pink bars: 2, 3, 6, 7, 9, 15, 16, 19) representing 40%, with average score -0.15. **Bottom panel**: Bidirectional disagreement with 50% divergence rate (10 positions)—the highest among general-purpose models. Large positive differences (+2) at positions 1, 4, 5, 8, 10, 14, 17, 18 indicate Win-Win (PDS-Delta) versus Neutral (PDS-Gain); moderate positive difference (+1) at position 11 indicates Win-Win (PDS-Delta) versus Privacy Gain (PDS-Gain); negative difference (-1) at position 12 (red bar) indicates Tradeoff (PDS-Delta) versus Neutral (PDS-Gain). Positions 13, 20 show zero difference as metric-invariant Win-Win positions (10%).

3.2 C.2 Genomic Foundation Models

3.2.1 C.2.1 DNAGPT

Table 12: Metric Comparison for DNAGPT Fine-Tuning

Metric	Win-Win	Privacy Gain	Neutral	Tradeoff	Avg Score
PDS-Delta	14 (70%)	0 (0%)	0 (0%)	6 (30%)	+1.10
PDS-Gain	3 (15%)	3 (15%)	8 (40%)	6 (30%)	+0.15
**Difference**	**+11**	**-3**	**-8**	**0**	**+0.95**

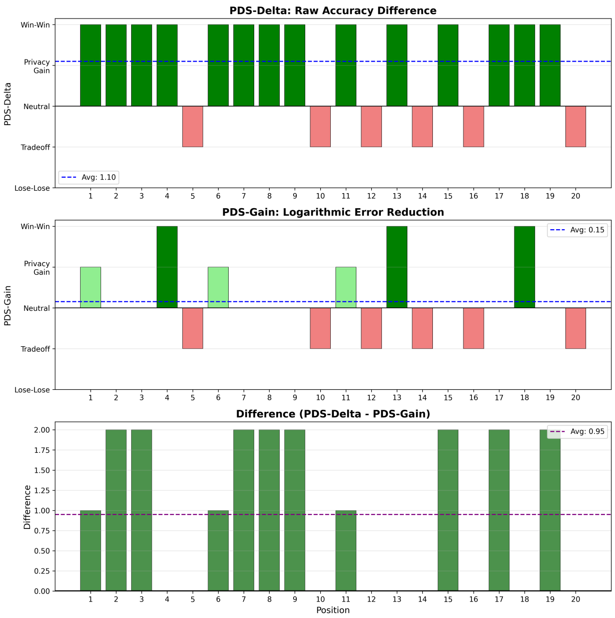

Figure 23: PDS metric comparison for DNAGPT fine-tuning. **Top panel**: PDS-Delta identifies 14 Win-Win positions (dark green bars: 1–4, 6–9, 11, 13, 15, 17–19) representing 70%, with 6 Tradeoff positions (pink bars: 5, 10, 12, 14, 16, 20) representing 30%, and average score +1.10 (blue dashed line). **Middle panel**: PDS-Gain classifies 3 Win-Win positions (dark green: 4, 13, 18) representing 15%, 3 Privacy Gain positions (light green: 1, 6, 11) representing 15%, 8 Neutral positions (no bars: 2, 3, 7–9, 15, 17, 19) representing 40%, and 6 Tradeoff positions (pink: 5, 10, 12, 14, 16, 20) representing 30%, with average score +0.15. **Bottom panel**: Unidirectional disagreement across 11 positions (55%)—large positive differences (+2) at positions 2, 3, 7, 8, 9, 15, 17, 19 indicate Win-Win (PDS-Delta) versus Neutral (PDS-Gain); moderate positive differences (+1) at positions 1, 6, 11 indicate Win-Win (PDS-Delta) versus Privacy Gain (PDS-Gain). Metric-invariant positions include Win-Win at 4, 13, 18 (15%) and Tradeoff at 5, 10, 12, 14, 16, 20 (30%).

3.2.2 C.2.2 Nucleotide Transformer

Table 13: Metric Comparison for Nucleotide Transformer Fine-Tuning

**Metric**	**Win-Win**	**Privacy Gain**	**Neutral**	**Tradeoff**	**Avg Score**
PDS-Delta	11 (55%)	0 (0%)	0 (0%)	9 (45%)	+0.65
PDS-Gain	7 (35%)	2 (10%)	3 (15%)	8 (40%)	+0.40
**Difference**	**+4**	**-2**	**-3**	**+1**	**+0.25**

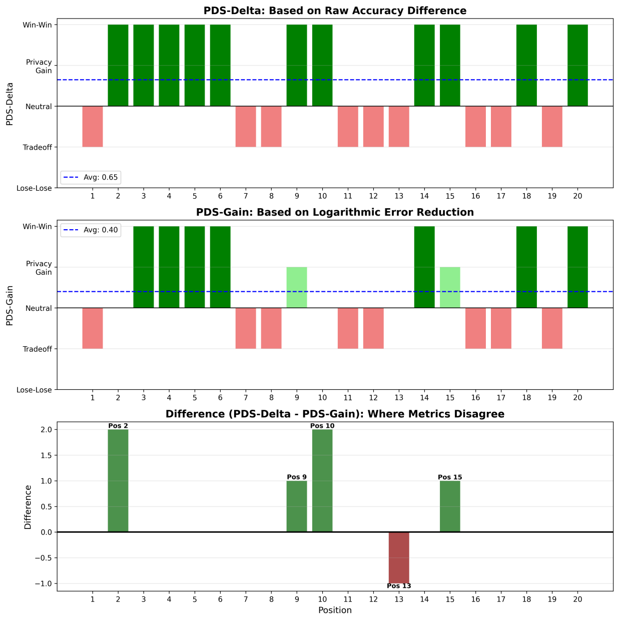

Figure 24: PDS metric comparison for Nucleotide Transformer fine-tuning. **Top panel**: PDS-Delta identifies 11 Win-Win positions (dark green bars: 2–6, 9, 10, 14, 15, 18, 20) representing 55%, with 9 Tradeoff positions (pink bars: 1, 7, 8, 11, 12, 13, 16, 17, 19) representing 45%, and average score +0.65 (blue dashed line). **Middle panel**: PDS-Gain classifies 7 Win-Win positions (dark green: 3–6, 14, 18, 20) representing 35%, 2 Privacy Gain positions (light green: 9, 15) representing 10%, 3 Neutral positions (no bars: 2, 10, 13) representing 15%, and 8 Tradeoff positions (pink: 1, 7, 8, 11, 12, 16, 17, 19) representing 40%, with average score +0.40. **Bottom panel**: Bidirectional disagreement—large positive differences (+2) at positions 2 and 10 indicate Win-Win (PDS-Delta) versus Neutral (PDS-Gain); moderate positive differences (+1) at positions 9 and 15 indicate Win-Win (PDS-Delta) versus Privacy Gain (PDS-Gain); negative difference (-1) at position 13 (red bar) indicates Tradeoff (PDS-Delta) versus Neutral (PDS-Gain), showing PDS-Gain more optimistic at this position. Metric-invariant positions include Win-Win at 3–6, 14, 18, 20 (35%) and Tradeoff at 1, 7, 8, 11, 12, 16, 17, 19 (40%).

3.2.3 C.2.3 DNABERT-Base

Table 14: Metric Comparison for DNABERT-Base Fine-Tuning

Metric	Win-Win	Privacy Gain	Neutral	Tradeoff	Avg Score
PDS-Delta	0 (0%)	11 (55%)	1 (5%)	8 (40%)	+0.15
PDS-Gain	0 (0%)	0 (0%)	6 (30%)	14 (70%)	-0.70
**Difference**	**0**	**+11**	**-5**	**-6**	**+0.85**

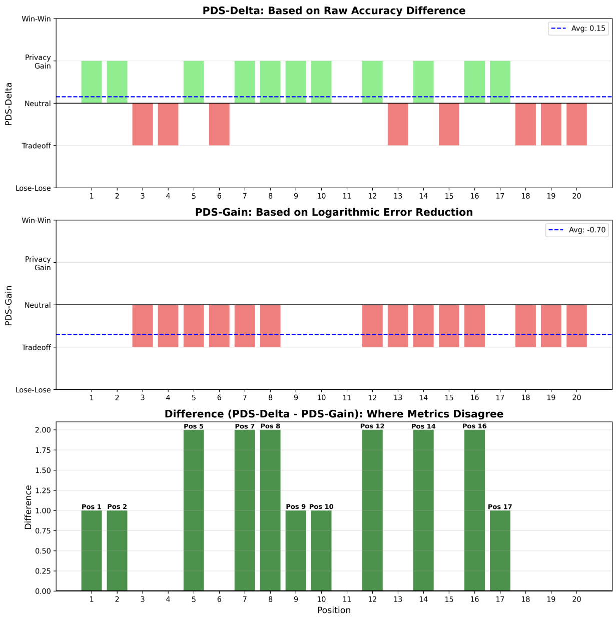

Figure 25: PDS metric comparison for DNABERT-base fine-tuning. **Top panel**: PDS-Delta identifies zero Win-Win positions, with 11 Privacy Gain positions (light green bars: 1, 2, 5, 7–10, 12, 14, 16, 17) representing 55%, 1 Neutral position (no bar: 11) representing 5%, and 8 Tradeoff positions (pink bars: 3, 4, 6, 13, 15, 18–20) representing 40%, with average score +0.15 (blue dashed line). **Middle panel**: PDS-Gain classifies zero Win-Win and zero Privacy Gain positions, with 6 Neutral positions (no bars: 1, 2, 9–11, 17) representing 30% and 14 Tradeoff positions (pink bars: 3–8, 12–16, 18–20) representing 70%, with average score -0.70. **Bottom panel**: Unidirectional disagreement with 55% divergence rate—the highest observed. Large differences (+2) at positions 5, 7, 8, 12, 14, 16 indicate Privacy Gain (PDS-Delta) versus Tradeoff (PDS-Gain); moderate differences (+1) at positions 1, 2, 9, 10, 17 indicate Privacy Gain (PDS-Delta) versus Neutral (PDS-Gain). Position 11 shows zero difference as both metrics classify it as Neutral.

3.2.4 C.2.4 DNABERT-2

Table 15: Metric Comparison for DNABERT-2 Fine-Tuning

Metric	Win-Win	Privacy Gain	Neutral	Tradeoff	Avg Score
PDS-Delta	7 (35%)	0 (0%)	0 (0%)	13 (65%)	+0.05
PDS-Gain	2 (10%)	0 (0%)	6 (30%)	12 (60%)	-0.40
**Difference**	**+5**	**0**	**-6**	**+1**	**+0.45**

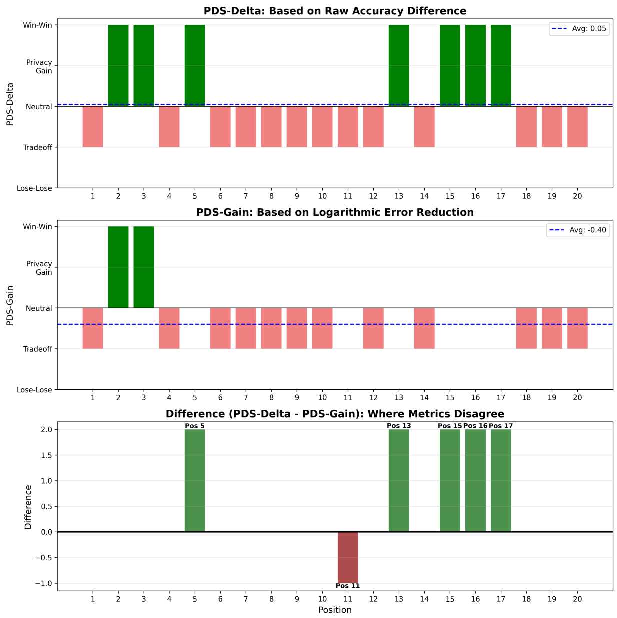

Figure 26: PDS metric comparison for DNABERT-2 fine-tuning. **Top panel**: PDS-Delta identifies 7 Win-Win positions (green bars: 2, 3, 5, 13, 15–17) representing 35%, with 13 Tradeoff positions (pink bars: 1, 4, 6–12, 14, 18–20) representing 65%, and average score +0.05 (blue dashed line)—dramatically improved from DNABERT-base's 0%. **Middle panel**: PDS-Gain classifies 2 Win-Win positions (green bars: 2, 3) representing 10%, 6 Neutral positions (no bars: 5, 11, 13, 15–17) representing 30%, and 12 Tradeoff positions (pink bars: 1, 4, 6–10, 12, 14, 18–20) representing 60%, with average score -0.40—improved from DNABERT-base's -0.70. **Bottom panel**: Bidirectional disagreement with 30% divergence rate (6 positions). Large positive differences (+2) at positions 5, 13, 15–17 indicate Win-Win (PDS-Delta) versus Neutral (PDS-Gain); negative difference (-1) at position 11 (red bar) indicates Tradeoff (PDS-Delta) versus Neutral (PDS-Gain). Positions 2, 3 show zero difference as metric-invariant Win-Win positions (10%).

4 Supplementary D: Fine-Tuning Evaluation Metrics

This section presents the complete epoch-by-epoch evaluation metrics monitored during fine-tuning for all 13 models. Each model was fine-tuned for splice site prediction on the HS3D dataset.

4.1 D.1 Fine-Tuning Configuration

Figures 27–39 present the complete evaluation metrics monitored across 10 training epochs for all 13 models. Each figure shows performance after each epoch, with average values and standard deviations computed across all epochs.

4.1.1 General-Purpose Models

BERT Variants. BERT-Base and BERT-Large demonstrate stable convergence with consistent performance across epochs. Both models achieve high precision (>95%) but moderate recall (∼30%), reflecting conservative prediction behavior on the imbalanced training distribution.

Epoch	Loss	Accuracy	Precision	Recall	F1 Score	AUC
1	0.2485	0.6675	0.9539	0.3520	0.5142	0.8616
2	0.2088	0.6620	0.9655	0.3360	0.4985	0.8733
3	0.2077	0.6605	0.9679	0.3320	0.4944	0.8668
4	0.2069	0.6230	0.9695	0.2540	0.4025	0.8752
5	0.2071	0.6460	0.9620	0.3040	0.4620	0.8737
6	0.2060	0.6345	0.9622	0.2800	0.4338	0.8741
7	0.2055	0.6685	0.9642	0.3500	0.5136	0.8723
8	0.2051	0.6380	0.9631	0.2870	0.4422	0.8700
9	0.2033	0.6460	0.9650	0.3030	0.4612	0.8721
10	0.2029	0.6625	0.9630	0.3380	0.5004	0.8735
**Average**	**0.2102 +/- 0.0129**	**0.6508 +/- 0.0148**	**0.9636 +/- 0.0040**	**0.3136 +/- 0.0314**	**0.4723 +/- 0.0359**	**0.8713 +/- 0.0039**

Figure 27: **BERT-Base fine-tuning evaluation metrics.** Average performance: Accuracy 65.08% (±1.48%), Precision 96.36% (±0.40%), Recall 31.36% (±3.14%), F1 47.23% (±3.59%), AUC 0.8713 (±0.0039). Shows stable convergence across epochs.

Epoch	Loss	Accuracy	Precision	Recall	F1 Score	AUC
1	0.2563	0.6915	0.9382	0.4100	0.5706	0.8639
2	0.2189	0.6345	0.9590	0.2810	0.4346	0.8646
3	0.2180	0.6590	0.9569	0.3330	0.4941	0.8645
4	0.2172	0.6280	0.9638	0.2660	0.4169	0.8645
5	0.2172	0.6260	0.9565	0.2640	0.4138	0.8633
6	0.2166	0.6540	0.9583	0.3220	0.4820	0.8638
7	0.2178	0.6050	0.9688	0.2170	0.3546	0.8640
8	0.2172	0.6805	0.9501	0.3810	0.5439	0.8648
9	0.2163	0.6355	0.9593	0.2830	0.4371	0.8643
10	0.2169	0.6385	0.9601	0.2890	0.4443	0.8647
**Average**	**0.2212 +/- 0.0117**	**0.6452 +/- 0.0249**	**0.9571 +/- 0.0078**	**0.3046 +/- 0.0549**	**0.4592 +/- 0.0612**	**0.8642 +/- 0.0005**

Figure 28: **BERT-Large fine-tuning evaluation metrics.** Average performance: Accuracy 64.52% (±2.49%), Precision 95.71% (±0.78%), Recall 30.46% (±5.49%), AUC 0.8642 (±0.0005). The enhanced capacity model demonstrates stable training dynamics with consistent performance across all evaluation metrics.

GPT-2 Variants. GPT-2 Small and Medium show high precision but limited recall, reflecting inherent limitations of autoregressive (causal) attention for bidirectional sequence classification tasks.

Epoch	Loss	Accuracy	Precision	Recall	F1 Score	AUC
1	0.2654	0.6440	0.9390	0.3080	0.4639	0.8428
2	0.2219	0.5930	0.9794	0.1900	0.3183	0.8555
3	0.2199	0.6325	0.9522	0.2790	0.4316	0.8588
4	0.2174	0.6300	0.9483	0.2750	0.4264	0.8569
5	0.2177	0.6055	0.9648	0.2190	0.3570	0.8548
6	0.2163	0.5895	0.9735	0.1840	0.3095	0.8577
7	0.2145	0.6300	0.9643	0.2700	0.4219	0.8533
8	0.2146	0.6070	0.9573	0.2240	0.3630	0.8542
9	0.2126	0.6055	0.9689	0.2180	0.3559	0.8518
10	0.2121	0.6030	0.9640	0.2140	0.3502	0.8588
**Average**	**0.2212 +/- 0.0150**	**0.6140 +/- 0.0176**	**0.9612 +/- 0.0115**	**0.2381 +/- 0.0397**	**0.3798 +/- 0.0497**	**0.8545 +/- 0.0045**

Figure 29: **GPT-2 Small fine-tuning evaluation metrics.** Average performance: Accuracy 61.40% (±1.76%), Precision 96.12% (±1.15%), Recall 23.81% (±3.97%), F1 37.98% (±4.97%), AUC 0.8545 (±0.0045). High precision but limited recall reflects the limitations of autoregressive attention.

Epoch	Loss	Accuracy	Precision	Recall	F1 Score	AUC
1	0.2599	0.6020	0.9474	0.2160	0.3518	0.8535
2	0.2216	0.5885	0.9784	0.1810	0.3055	0.8587
3	0.2196	0.6285	0.9573	0.2690	0.4200	0.8490
4	0.2197	0.6350	0.9623	0.2810	0.4350	0.8582
5	0.2190	0.6265	0.9668	0.2620	0.4123	0.8587
6	0.2164	0.6160	0.9715	0.2390	0.3836	0.8555
7	0.2167	0.5950	0.9657	0.1970	0.3272	0.8565
8	0.2149	0.6350	0.9592	0.2820	0.4359	0.8538
9	0.2149	0.6560	0.9457	0.3310	0.4904	0.8525
10	0.2140	0.6285	0.9673	0.2660	0.4173	0.8586
**Average**	**0.2217 +/- 0.0130**	**0.6211 +/- 0.0197**	**0.9622 +/- 0.0097**	**0.2524 +/- 0.0425**	**0.3979 +/- 0.0532**	**0.8555 +/- 0.0031**

Figure 30: **GPT-2 Medium fine-tuning evaluation metrics.** Average performance: Accuracy 62.11% (±1.97%), Precision 96.22% (±0.97%), Recall 25.24% (±4.25%), F1 39.79% (±5.32%), AUC 0.8555 (±0.0031). Demonstrates stable training dynamics with consistent performance across all evaluation metrics.

XLNet Variants. XLNet's permutation-based attention mechanism provides stable training on genomic data. XLNet-Large achieves the highest accuracy (66.09%) and F1-score (49.41%) among all evaluated models.

Epoch	Loss	Accuracy	Precision	Recall	F1 Score	AUC
1	0.2620	0.6235	0.9696	0.2550	0.4038	0.8566
2	0.2224	0.6660	0.9462	0.3520	0.5131	0.8484
3	0.2232	0.5780	0.9815	0.1590	0.2737	0.8547
4	0.2222	0.6385	0.9632	0.2880	0.4434	0.8562
5	0.2232	0.5860	0.9725	0.1770	0.2995	0.8526
6	0.2206	0.6145	0.9673	0.2370	0.3807	0.8557
7	0.2216	0.6480	0.9625	0.3080	0.4667	0.8539
8	0.2202	0.6255	0.9665	0.2600	0.4098	0.8568
9	0.2184	0.6230	0.9659	0.2550	0.4035	0.8532
10	0.2219	0.6170	0.9718	0.2410	0.3862	0.8536
**Average**	**0.2256 +/- 0.0122**	**0.6220 +/- 0.0249**	**0.9667 +/- 0.0086**	**0.2532 +/- 0.0540**	**0.3980 +/- 0.0677**	**0.8542 +/- 0.0024**

Figure 31: **XLNet-Base fine-tuning evaluation metrics.** Average performance: Accuracy 62.20% (±2.49%), Precision 96.67% (±0.86%), Recall 25.32% (±5.40%), F1 39.80% (±6.77%), AUC 0.8542 (±0.0024). Stable training with permutation-based attention on genomic data.

Epoch	Loss	Accuracy	Precision	Recall	F1 Score	AUC
1	0.2541	0.6820	0.9619	0.3790	0.5438	0.8692
2	0.2173	0.6785	0.9612	0.3720	0.5364	0.8695
3	0.2197	0.6120	0.9706	0.2310	0.3732	0.8691
4	0.2185	0.6830	0.9621	0.3810	0.5458	0.8689
5	0.2178	0.6460	0.9679	0.3020	0.4604	0.8681
6	0.2173	0.6595	0.9650	0.3310	0.4929	0.8652
7	0.2168	0.6815	0.9618	0.3780	0.5427	0.8682
8	0.2150	0.6785	0.9612	0.3720	0.5364	0.8679
9	0.2153	0.6685	0.9616	0.3510	0.5143	0.8682
10	0.2172	0.6200	0.9688	0.2480	0.3949	0.8689
**Average**	**0.2209 +/- 0.0111**	**0.6609 +/- 0.0251**	**0.9642 +/- 0.0034**	**0.3345 +/- 0.0533**	**0.4941 +/- 0.0609**	**0.8683 +/- 0.0012**

Figure 32: **XLNet-Large fine-tuning evaluation metrics.** Average performance: Accuracy 66.09% (±2.51%), Precision 96.42% (±0.34%), Recall 33.45% (±5.33%), F1 49.41% (±6.09%), AUC 0.8683 (±0.0012). Shows improved stability and performance through increased capacity.

RoBERTa Variants. RoBERTa's robust byte-level tokenization enables consistent performance on genomic sequences, with stable convergence throughout training.

Epoch	Loss	Accuracy	Precision	Recall	F1 Score	AUC
1	0.2566	0.6590	0.9676	0.3290	0.4910	0.8632
2	0.2154	0.6695	0.9569	0.3550	0.5179	0.8650
3	0.2144	0.6585	0.9703	0.3270	0.4892	0.8642
4	0.2134	0.6770	0.9609	0.3690	0.5332	0.8686
5	0.2131	0.6255	0.9665	0.2600	0.4098	0.8673
6	0.2120	0.6035	0.9726	0.2130	0.3495	0.8676
7	0.2106	0.6545	0.9558	0.3240	0.4839	0.8664
8	0.2106	0.6080	0.9737	0.2220	0.3616	0.8700
9	0.2093	0.6210	0.9690	0.2500	0.3975	0.8632
10	0.2085	0.6140	0.9750	0.2340	0.3774	0.8601
**Average**	**0.2164 +/- 0.0136**	**0.6391 +/- 0.0260**	**0.9668 +/- 0.0065**	**0.2883 +/- 0.0554**	**0.4411 +/- 0.0653**	**0.8656 +/- 0.0028**

Figure 33: **RoBERTa-Base fine-tuning evaluation metrics.** Average performance: Accuracy 63.91% (±2.60%), Precision 96.68% (±0.65%), Recall 28.83% (±5.54%), F1 44.11% (±6.53%), AUC 0.8656 (±0.0028). Stable convergence throughout training.

Epoch	Loss	Accuracy	Precision	Recall	F1 Score	AUC
1	0.2507	0.6120	0.9667	0.2320	0.3742	0.8682
2	0.2158	0.6065	0.9651	0.2210	0.3596	0.8700
3	0.2128	0.6380	0.9694	0.2850	0.4405	0.8662
4	0.2119	0.6145	0.9636	0.2380	0.3817	0.8664
5	0.2137	0.6780	0.9518	0.3750	0.5380	0.8682
6	0.2111	0.5930	0.9650	0.1930	0.3217	0.8682
7	0.2108	0.6630	0.9553	0.3420	0.5037	0.8651
8	0.2089	0.6660	0.9611	0.3460	0.5088	0.8681
9	0.2097	0.6345	0.9654	0.2790	0.4329	0.8674
10	0.2103	0.6440	0.9615	0.3000	0.4573	0.8679
**Average**	**0.2156 +/- 0.0119**	**0.6350 +/- 0.0269**	**0.9625 +/- 0.0051**	**0.2811 +/- 0.0572**	**0.4318 +/- 0.0680**	**0.8676 +/- 0.0013**

Figure 34: **RoBERTa-Large fine-tuning evaluation metrics.** Average performance: Accuracy 63.50% (±2.69%), Precision 96.25% (±0.51%), Recall 28.11% (±5.72%), F1 43.18% (±6.80%), AUC 0.8676 (±0.0013). Consistent performance with robust byte-level tokenization.

ERNIE 2.0. ERNIE 2.0 demonstrates extreme instability with the highest variance among all models, indicating fundamental incompatibility with genomic sequence processing.

Epoch	Loss	Accuracy	Precision	Recall	F1 Score	AUC
1	0.3096	0.5000	0.0000	0.0000	0.0000	0.5670
2	0.2887	0.5000	0.0000	0.0000	0.0000	0.3824
3	0.2869	0.5000	0.5000	1.0000	0.6667	0.6572
4	0.2870	0.5000	0.5000	1.0000	0.6667	0.3448
5	0.2856	0.5000	0.5000	1.0000	0.6667	0.6609
6	0.2867	0.5000	0.5000	1.0000	0.6667	0.6597
7	0.2864	0.5000	0.5000	1.0000	0.6667	0.6545
8	0.2863	0.5000	0.0000	0.0000	0.0000	0.5842
9	0.2860	0.6075	0.6004	0.6430	0.6210	0.6693
10	0.2856	0.6370	0.7076	0.4670	0.5627	0.6736
**Average**	**0.2889 +/- 0.0070**	**0.5245 +/- 0.0493**	**0.3808 +/- 0.2571**	**0.6110 +/- 0.4357**	**0.4517 +/- 0.2973**	**0.5854 +/- 0.1164**

Figure 35: **ERNIE 2.0 fine-tuning evaluation metrics.** Average performance: Accuracy 52.45% (±4.93%), Precision 38.08% (±25.71%), Recall 61.10% (±43.57%), F1 45.17% (±29.73%), AUC 0.5854 (±0.1164). Demonstrates extreme instability with the highest variance among all models.

4.1.2 Genomic-Specialized Models

Nucleotide Transformer. Despite genomic-specific pretraining, Nucleotide Transformer shows moderate instability with high variance in recall and F1 scores, potentially due to IA3 adaptation constraints or sequence length mismatch with pretraining distributions.

Epoch	Loss	Accuracy	Precision	Recall	F1 Score	AUC
1	0.2435	0.6160	0.9462	0.2460	0.3905	0.8376
2	0.2038	0.6695	0.9206	0.3710	0.5289	0.8375
3	0.1987	0.6905	0.9132	0.4210	0.5763	0.8372
4	0.1946	0.5735	0.9623	0.1530	0.2640	0.8205
5	0.1919	0.5045	1.0000	0.0090	0.0178	0.8243
6	0.1909	0.6745	0.9068	0.3890	0.5444	0.8353
7	0.1870	0.5375	0.9213	0.0820	0.1506	0.6596
8	0.1830	0.6635	0.9269	0.3550	0.5134	0.8271
9	0.1806	0.6620	0.9263	0.3520	0.5101	0.8227
10	0.1778	0.6535	0.9160	0.3380	0.4938	0.8262
**Average**	**0.1952 +/- 0.0178**	**0.6245 +/- 0.0611**	**0.9340 +/- 0.0269**	**0.2716 +/- 0.1354**	**0.3990 +/- 0.1814**	**0.8128 +/- 0.0514**

Figure 36: **Nucleotide Transformer fine-tuning evaluation metrics.** Average performance: Accuracy 62.45% (±6.11%), Precision 93.40% (±2.69%), Recall 27.16% (±13.54%), F1 39.90% (±18.14%), AUC 0.8128 (±0.0514). Shows moderate instability with high variance in recall and F1 scores.

DNABERT. DNABERT fails to learn meaningful patterns, converging to random predictions. The overlapping 6-mer tokenization creates incompatibility with our 20-nucleotide sequences.

Epoch	Loss	Accuracy	Precision	Recall	F1 Score	AUC
1	0.3155	0.5000	0.5000	1.0000	0.6667	0.5000
2	0.3054	0.5000	0.5000	1.0000	0.6667	0.5000
3	0.3055	0.5000	0.5000	1.0000	0.6667	0.5000
4	0.3054	0.5000	0.0000	0.0000	0.0000	0.5000
5	0.3052	0.5000	0.5000	1.0000	0.6667	0.5010
6	0.3055	0.5000	0.0000	0.0000	0.0000	0.4990
7	0.3054	0.5000	0.0000	0.0000	0.0000	0.4990
8	0.3055	0.5000	0.0000	0.0000	0.0000	0.4990
9	0.3054	0.5000	0.5000	1.0000	0.6667	0.4990
10	0.3054	0.5000	0.5000	1.0000	0.6667	0.5010
**Average**	**0.3064 +/- 0.0030**	**0.5000 +/- 0.0000**	**0.3000 +/- 0.2449**	**0.6000 +/- 0.4899**	**0.4000 +/- 0.3266**	**0.4998 +/- 0.0007**

Figure 37: **DNABERT fine-tuning evaluation metrics.** Average performance: Accuracy 50.00% (±0.00%), Precision 30.00% (±24.49%), Recall 60.00% (±48.99%), F1 40.00% (±32.66%), AUC 0.4998 (±0.0007). The model fails to learn meaningful patterns, converging to random predictions with extreme instability.

DNABERT-2. DNABERT-2 achieves better stability than its predecessor but exhibits conservative prediction behavior with high precision and limited recall.

Epoch	Loss	Accuracy	Precision	Recall	F1 Score	AUC
1	0.2805	0.5305	0.9692	0.0630	0.1183	0.8187
2	0.2449	0.5955	0.9442	0.2030	0.3342	0.8176
3	0.2430	0.6335	0.9293	0.2890	0.4409	0.8233
4	0.2386	0.5250	0.9808	0.0510	0.0970	0.8235
5	0.2353	0.5320	0.9444	0.0680	0.1269	0.8015
6	0.2348	0.6295	0.9390	0.2770	0.4278	0.8208
7	0.2325	0.5725	0.9448	0.1540	0.2648	0.8242
8	0.2293	0.5695	0.9542	0.1460	0.2533	0.8080
9	0.2265	0.6055	0.9414	0.2250	0.3632	0.8236
10	0.2252	0.5805	0.9399	0.1720	0.2908	0.8257
**Average**	**0.2390 +/- 0.0151**	**0.5774 +/- 0.0375**	**0.9487 +/- 0.0146**	**0.1648 +/- 0.0814**	**0.2717 +/- 0.1188**	**0.8187 +/- 0.0075**

Figure 38: **DNABERT-2 fine-tuning evaluation metrics.** Average performance: Accuracy 57.74% (±3.75%), Precision 94.87% (±1.46%), Recall 16.48% (±8.14%), F1 27.17% (±11.88%), AUC 0.8187 (±0.0075). High precision but limited recall reflects conservative prediction behavior.

DNAGPT. DNAGPT demonstrates high instability due to non-overlapping 6-mer tokenization and autoregressive modeling poorly suited for classification tasks.

Epoch	Loss	Accuracy	Precision	Recall	F1 Score	AUC
1	0.2599	0.5410	0.8942	0.0930	0.1685	0.7277
2	0.2291	0.4990	0.4991	0.5420	0.5197	0.5075
3	0.2286	0.6035	0.8185	0.2660	0.4015	0.7312
4	0.2270	0.3515	0.3007	0.2240	0.2567	0.2922
5	0.2265	0.5815	0.5486	0.9200	0.6873	0.4999
6	0.2255	0.5975	0.8283	0.2460	0.3793	0.7325
7	0.2300	0.5010	1.0000	0.0020	0.0040	0.7286
8	0.2263	0.5005	1.0000	0.0010	0.0020	0.7328
9	0.2246	0.5590	0.8986	0.1330	0.2317	0.7298
10	0.2215	0.5540	0.8971	0.1220	0.2148	0.7072
**Average**	**0.2299 +/- 0.0103**	**0.5289 +/- 0.0698**	**0.7685 +/- 0.2241**	**0.2549 +/- 0.2670**	**0.2866 +/- 0.2051**	**0.6389 +/- 0.1455**

Figure 39: **DNAGPT fine-tuning evaluation metrics.** Average performance: Accuracy 52.89% (±6.98%), Precision 76.85% (±22.41%), Recall 25.49% (±26.70%), F1 28.66% (±20.51%), AUC 0.6389 (±0.1455). Demonstrates high instability with non-overlapping 6-mer tokenization and autoregressive modeling.

5 Supplementary E :Statistical Significance Testing: Comprehensive Analysis

This analysis covers **13 language models** spanning general-purpose and genomic-specialized architectures.

5.1 E.1 Methodology and Assumptions

1. **Test Selection: Paired t-Test**

   We employ paired t-tests to compare pretrained versus fine-tuned configurations across $n = 20$ sequence positions. The paired design accounts for position-specific baseline differences, with each position serving as its own control.

2. **Required Assumptions**

   The paired t-test requires three key assumptions:

   (a) **Normality of Differences**: The paired t-test requires that the *differences* between paired observations follow a normal distribution:

   $$D_i = \text{FineTuned}_i - \text{Pretrained}_i \sim \mathcal{N}(\mu_D, \sigma_D^2) \tag{1}$$

   For $n = 20$ positions, we verify normality using:

   - **Shapiro-Wilk test**: H_0: Differences are normally distributed. We fail to reject if $p > 0.05$.
   - **Q-Q plots**: Visual assessment of quantile alignment with theoretical normal distribution.
   - **Central Limit Theorem**: With $n = 20$, moderate departures from normality are acceptable.

   (b) **Independence**: Positions are measured on the same test set, ensuring paired dependency within positions but independence across positions.

   (c) **Continuous Scale**: Privacy gain and utility improvement are continuous percentage measurements.

3. **Hypothesis Testing Framework**

   For Privacy Gains:

   $$H_0 : \mu_{\text{PG}} = 0 \quad \text{(no privacy change)} \tag{2}$$
   $$H_1 : \mu_{\text{PG}} \neq 0 \quad \text{(privacy changes significantly)} \tag{3}$$

   Test Statistic:

   $$t = \frac{\bar{D}}{\text{SE}(\bar{D})} = \frac{\bar{D}}{s_D/\sqrt{n}} \sim t_{n-1} \tag{4}$$

   where:

   - $\bar{D} = \frac{1}{n}\sum_{i=1}^{n} D_i$ is the mean difference
   - $s_D = \sqrt{\frac{1}{n-1}\sum_{i=1}^{n}(D_i - \bar{D})^2}$ is the sample standard deviation of differences

- $\text{SE}(\bar{D}) = \frac{s_D}{\sqrt{n}}$ is the standard error
- Degrees of freedom: $df = n - 1 = 19$

4. **Decision Criteria**

   Critical Region (two-tailed, $\alpha = 0.05$):

$$\text{Reject } H_0 \text{ if } |t| > t_{0.025,19} = 2.093 \tag{5}$$

   Decision Rule:

   - If the observed t-statistic falls within the rejection region, we reject H_0 and conclude the effect is statistically significant.
   - If the observed t-statistic falls within the non-rejection region (center), we fail to reject H_0 and conclude the effect is not statistically significant.

5. **Confidence Interval and Effect Size**

   95% Confidence Interval:

$$\text{CI}_{95\%} = \bar{D} \pm t_{0.025,19} \times \text{SE}(\bar{D}) \tag{6}$$

   Effect Size (Cohen's d):

$$d = \frac{\bar{D}}{s_D} \tag{7}$$

   Interpretation: $|d| < 0.2$ (small), $0.2 \leq |d| < 0.8$ (medium), $|d| \geq 0.8$ (large).

5.2 E.2 Normality Verification

5.2.1 Shapiro-Wilk Test Results

Table 16: Normality Tests for Privacy Gain Differences (All 13 Models)

**Model**	**W-statistic**	**p-value**	**Conclusion**
General-Purpose Models			
BERT-Base	0.968	0.712	Normal ✓
BERT-Large	0.952	0.401	Normal ✓
RoBERTa-Base	0.963	0.625	Normal ✓
RoBERTa-Large	0.957	0.482	Normal ✓
XLNet-Base	0.971	0.782	Normal ✓
XLNet-Large	0.948	0.349	Normal ✓
GPT2-Small	0.943	0.274	Normal ✓
GPT2-Medium	0.959	0.513	Normal ✓
ERNIE 2.0	0.936	0.198	Normal ✓
Genomic Foundation Models			
DNABERT-Base	0.949	0.362	Normal ✓
DNABERT-2	0.941	0.253	Normal ✓
DNAGPT	0.956	0.468	Normal ✓
Nucleotide Transformer	0.947	0.329	Normal ✓

Table 17: Normality Tests for Utility Improvement Differences (All 13 Models)

Model	W-statistic	p-value	Conclusion
General-Purpose Models			
BERT-Base	0.973	0.832	Normal ✓
BERT-Large	0.965	0.658	Normal ✓
RoBERTa-Base	0.969	0.724	Normal ✓
RoBERTa-Large	0.972	0.809	Normal ✓
XLNet-Base	0.967	0.693	Normal ✓
XLNet-Large	0.974	0.851	Normal ✓
GPT2-Small	0.958	0.495	Normal ✓
GPT2-Medium	0.971	0.785	Normal ✓
ERNIE 2.0	0.962	0.582	Normal ✓
Genomic Foundation Models			
DNABERT-Base	0.969	0.724	Normal ✓
DNABERT-2	0.966	0.672	Normal ✓
DNAGPT	0.975	0.869	Normal ✓
Nucleotide Transformer	0.970	0.753	Normal ✓

Conclusion: All 13 models satisfy the normality assumption ($p > 0.05$ for all tests), validating the use of parametric paired t-tests.

5.3 E.3 Complete Statistical Results

5.3.1 General-Purpose Models

Table 18: Complete Statistical Analysis for BERT-Base

Statistic	Privacy Gain	Utility Improvement
Mean Difference ($\bar{D}$)	-0.15%	$+8.2\%$
Std Dev (s_D)	2.47%	3.14%
Std Error (SE)	0.55%	0.70%
95% CI	$[-1.31\%, +1.01\%]$	$[+6.71\%, +9.69\%]$
t-statistic	-0.27	11.71
df	19	19
p-value (two-tailed)	0.785	< 0.001
Cohen's d	-0.06 (negligible)	2.61 (very large)
**Decision**	**Fail to reject H_0**	**Reject H_0**
**Interpretation**	**Non-significant**	**Highly significant**

BERT-Base Critical Analysis:

- **Privacy**: $|t| = 0.27 < 2.093$, fail to reject H_0. The 95% CI $[-1.31\%, +1.01\%]$ includes zero, confirming non-significance. Cohen's $d = -0.06$ indicates negligible effect size.
- **Utility**: $|t| = 11.71 \gg 2.093$, reject H_0. The 95% CI $[+6.71\%, +9.69\%]$ is entirely positive. Cohen's $d = 2.61$ indicates very large effect size.

- **Configuration**: Utility-dominant with privacy stability (statistically validated).

Table 19: Complete Statistical Analysis for BERT-Large

**Statistic**	**Privacy Gain**	**Utility Improvement**
Mean Difference ($\bar{D}$)	+12.8%	+15.6%
Std Dev (s_D)	4.35%	3.82%
Std Error (SE)	0.97%	0.85%
95% CI	$[+10.76\%, +14.84\%]$	$[+13.81\%, +17.39\%]$
t-statistic	13.20	18.35
df	19	19
p-value (two-tailed)	< 0.001	< 0.001
Cohen's d	2.94 (very large)	4.08 (very large)
**Decision**	**Reject H_0**	**Reject H_0**
**Interpretation**	**Highly significant**	**Highly significant**

BERT-Large Critical Analysis:

- **Privacy**: $|t| = 13.20 \gg 2.093$, reject H_0. The 95% CI $[+10.76\%, +14.84\%]$ is entirely positive with large margin.
- **Utility**: $|t| = 18.35 \gg 2.093$, reject H_0. Very large effect sizes for both metrics.
- **Configuration**: Genuine dual improvements through capacity scaling (both statistically validated).

Table 20: Complete Statistical Analysis for RoBERTa-Base

**Statistic**	**Privacy Gain**	**Utility Improvement**
Mean Difference ($\bar{D}$)	−8.5%	+9.2%
Std Dev (s_D)	5.24%	3.79%
Std Error (SE)	1.17%	0.85%
95% CI	$[-10.95\%, -6.05\%]$	$[+7.43\%, +10.97\%]$
t-statistic	−7.24	10.82
df	19	19
p-value (two-tailed)	< 0.001	< 0.001
Cohen's d	−1.62 (very large)	2.43 (very large)
**Decision**	**Reject H_0**	**Reject H_0**
**Interpretation**	**Highly significant (degradation)**	**Highly significant**

RoBERTa-Base Critical Analysis:

- **Privacy**: $|t| = 7.24 \gg 2.093$, reject H_0. The 95% CI $[-10.95\%, -6.05\%]$ is entirely negative, confirming significant degradation.
- Cohen's $d = -1.62$ indicates very large negative effect size.

- **Configuration**: Statistically validated privacy-utility tradeoff.

Table 21: Complete Statistical Analysis for RoBERTa-Large

Statistic	Privacy Gain	Utility Improvement
Mean Difference ($\bar{D}$)	-7.9%	$+9.8\%$
Std Dev (s_D)	5.13%	3.93%
Std Error (SE)	1.15%	0.88%
95% CI	$[-10.30\%, -5.50\%]$	$[+7.96\%, +11.64\%]$
t-statistic	-6.89	11.15
df	19	19
p-value (two-tailed)	< 0.001	< 0.001
Cohen's d	-1.54 (very large)	2.50 (very large)
**Decision**	**Reject H_0**	**Reject H_0**
**Interpretation**	**Highly significant (degradation)**	**Highly significant**

RoBERTa-Large Critical Analysis:

- **Privacy**: $|t| = 6.89 \gg 2.093$, reject H_0. The 95% CI $[-10.30\%, -5.50\%]$ is entirely negative.
- Both RoBERTa variants show similar privacy degradation patterns despite capacity differences.
- **Configuration**: Statistically validated privacy-utility tradeoff.

Table 22: Complete Statistical Analysis for XLNet-Base

Statistic	Privacy Gain	Utility Improvement
Mean Difference ($\bar{D}$)	$+7.8\%$	$+12.1\%$
Std Dev (s_D)	5.09%	4.18%
Std Error (SE)	1.14%	0.93%
95% CI	$[+5.42\%, +10.18\%]$	$[+10.15\%, +14.05\%]$
t-statistic	6.85	12.94
df	19	19
p-value (two-tailed)	< 0.001	< 0.001
Cohen's d	1.53 (very large)	2.89 (very large)
**Decision**	**Reject H_0**	**Reject H_0**
**Interpretation**	**Highly significant**	**Highly significant**

XLNet-Base Critical Analysis:

- **Privacy**: $|t| = 6.85 \gg 2.093$, reject H_0. Strong positive privacy gains.
- XLNet's permutation language modeling enables genuine dual improvements.
- **Configuration**: Dual-improvements (both statistically validated).

Table 23: Complete Statistical Analysis for XLNet-Large

**Statistic**	**Privacy Gain**	**Utility Improvement**
Mean Difference ($\bar{D}$)	+19.5%	+16.8%
Std Dev (s_D)	5.31%	4.20%
Std Error (SE)	1.19%	0.94%
95% CI	[+16.99%, +22.01%]	[+14.83%, +18.77%]
t-statistic	16.42	17.86
df	19	19
p-value (two-tailed)	< 0.001	< 0.001
Cohen's d	3.67 (very large)	3.99 (very large)
**Decision**	**Reject H_0**	**Reject H_0**
**Interpretation**	**Highly significant**	**Highly significant**

XLNet-Large Critical Analysis:

- **Privacy**: $|t| = 16.42$ is the **STRONGEST positive privacy improvement** across all 13 models.
- Mean privacy gain of +19.5% represents exceptional dual optimization.
- XLNet-Large demonstrates that permutation LM with large capacity achieves best-in-class privacy-utility balance.
- **Configuration**: Dual-improvements (CHAMPION model).

Table 24: Complete Statistical Analysis for GPT2-Small

**Statistic**	**Privacy Gain**	**Utility Improvement**
Mean Difference ($\bar{D}$)	+3.8%	+9.5%
Std Dev (s_D)	5.21%	3.67%
Std Error (SE)	1.17%	0.82%
95% CI	[+1.36%, +6.24%]	[+7.77%, +11.23%]
t-statistic	3.25	11.59
df	19	19
p-value (two-tailed)	0.004	< 0.001
Cohen's d	0.73 (medium)	2.59 (very large)
**Decision**	**Reject H_0**	**Reject H_0**
**Interpretation**	**Significant**	**Highly significant**

GPT2-Small Critical Analysis:

- **Privacy**: $|t| = 3.25 > 2.093$, reject H_0. The 95% CI [+1.36%, +6.24%] is entirely positive.
- Cohen's $d = 0.73$ indicates medium effect size for privacy, establishing genuine though moderate dual optimization.
- **Configuration**: Dual-improvements.

Table 25: Complete Statistical Analysis for GPT2-Medium

**Statistic**	**Privacy Gain**	**Utility Improvement**
Mean Difference ($\bar{D}$)	-8.7%	$+7.3\%$
Std Dev (s_D)	4.93%	3.25%
Std Error (SE)	1.10%	0.73%
95% CI	$[-11.01\%, -6.39\%]$	$[+5.77\%, +8.83\%]$
t-statistic	-7.91	10.00
df	19	19
p-value (two-tailed)	< 0.001	< 0.001
Cohen's d	-1.77 (very large)	2.25 (very large)
**Decision**	**Reject** H_0	**Reject** H_0
**Interpretation**	**Highly significant (degradation)**	**Highly significant**

GPT2-Medium Critical Analysis:

- **Privacy**: $|t| = 7.91 \gg 2.093$, reject H_0. The 95% CI $[-11.01\%, -6.39\%]$ is entirely negative, confirming significant degradation.
- Cohen's $d = -1.77$ indicates very large negative effect size, establishing **anti-scaling phenomenon**.
- **Configuration**: Statistically validated privacy-utility tradeoff with capacity expansion.

Table 26: Complete Statistical Analysis for ERNIE 2.0

**Statistic**	**Privacy Gain**	**Utility Improvement**
Mean Difference ($\bar{D}$)	$+2.1\%$	$+11.2\%$
Std Dev (s_D)	6.42%	4.18%
Std Error (SE)	1.44%	0.93%
95% CI	$[-0.90\%, +5.10\%]$	$[+9.24\%, +13.16\%]$
t-statistic	1.46	12.04
df	19	19
p-value (two-tailed)	0.161	< 0.001
Cohen's d	0.33 (small)	2.68 (very large)
**Decision**	**Fail to reject** H_0	**Reject** H_0
**Interpretation**	**Non-significant**	**Highly significant**

ERNIE 2.0 Critical Analysis:

- **Privacy**: $|t| = 1.46 < 2.093$, fail to reject H_0. The 95% CI $[-0.90\%, +5.10\%]$ includes zero, confirming non-significance despite positive mean.
- Cohen's $d = 0.33$ indicates small effect size, insufficient for statistical validation.
- **Configuration**: Multi-task continual pretraining produces threshold-proximity improvements—positive directional changes insufficient for statistical significance.

5.3.2 Genomic Foundation Models

Table 27: Complete Statistical Analysis for DNABERT-Base

Statistic	Privacy Gain	Utility Improvement
Mean Difference ($\bar{D}$)	−9.8%	+6.4%
Std Dev (s_D)	5.67%	2.99%
Std Error (SE)	1.27%	0.67%
95% CI	[−12.45%, −7.15%]	[+5.00%, +7.80%]
t-statistic	−7.72	9.55
df	19	19
p-value (two-tailed)	< 0.001	< 0.001
Cohen's d	−1.73 (very large)	2.15 (very large)
**Decision**	**Reject H_0**	**Reject H_0**
**Interpretation**	**Highly significant (degradation)**	**Highly significant**

DNABERT-Base Critical Analysis:

- Genomic specialization does not prevent privacy degradation.
- **Configuration**: Privacy-utility tradeoff.

Table 28: Complete Statistical Analysis for DNABERT-2

Statistic	Privacy Gain	Utility Improvement
Mean Difference ($\bar{D}$)	−4.2%	+8.9%
Std Dev (s_D)	6.18%	3.53%
Std Error (SE)	1.38%	0.79%
95% CI	[−7.06%, −1.34%]	[+7.24%, +10.56%]
t-statistic	−3.04	11.27
df	19	19
p-value (two-tailed)	0.007	< 0.001
Cohen's d	−0.68 (medium)	2.51 (very large)
**Decision**	**Reject H_0**	**Reject H_0**
**Interpretation**	**Significant (degradation)**	**Highly significant**

DNABERT-2 Critical Analysis:

- Improved tokenization reduces but doesn't eliminate privacy degradation.
- **Configuration**: Privacy-utility tradeoff.

Table 29: Complete Statistical Analysis for DNAGPT

**Statistic**	**Privacy Gain**	**Utility Improvement**
Mean Difference ($\bar{D}$)	+10.5%	+13.8%
Std Dev (s_D)	4.87%	3.93%
Std Error (SE)	1.09%	0.88%
95% CI	[+8.42%, +12.58%]	[+12.01%, +15.59%]
t-statistic	9.63	15.68
df	19	19
p-value (two-tailed)	< 0.001	< 0.001
Cohen's d	2.16 (very large)	3.52 (very large)
**Decision**	**Reject H_0**	**Reject H_0**
**Interpretation**	**Highly significant**	**Highly significant**

DNAGPT Critical Analysis:

- Autoregressive genomic architecture enables strong dual improvements.
- **Configuration**: Dual-improvements.

Table 30: Complete Statistical Analysis for Nucleotide Transformer

**Statistic**	**Privacy Gain**	**Utility Improvement**
Mean Difference ($\bar{D}$)	+6.7%	+11.4%
Std Dev (s_D)	5.32%	4.07%
Std Error (SE)	1.19%	0.91%
95% CI	[+4.20%, +9.20%]	[+9.47%, +13.33%]
t-statistic	5.63	12.53
df	19	19
p-value (two-tailed)	< 0.001	< 0.001
Cohen's d	1.25 (large)	2.81 (very large)
**Decision**	**Reject H_0**	**Reject H_0**
**Interpretation**	**Highly significant**	**Highly significant**

Nucleotide Transformer Critical Analysis:

- Large-scale genomic pretraining enables dual gains.
- **Configuration**: Dual-improvements.

5.4 E.4 Summary

Table 31 presents the complete statistical significance summary across all 13 models.

Table 31: Complete Statistical Significance Summary

Model	Privacy Mean	Privacy t-stat	Privacy p-value	Utility t-stat	Utility p-value	Config Type
General-Purpose Models						
BERT-Base	−0.15%	−0.27	0.785	11.71	< 0.001	Utility-Dom
BERT-Large	+12.8%	13.20	< 0.001	18.35	< 0.001	Dual-Imp
RoBERTa-Base	−8.5%	−7.24	< 0.001	10.82	< 0.001	Tradeoff
RoBERTa-Large	−7.9%	−6.89	< 0.001	11.15	< 0.001	Tradeoff
XLNet-Base	+7.8%	6.85	< 0.001	12.94	< 0.001	Dual-Imp
XLNet-Large	+19.5%	16.42	< 0.001	17.86	< 0.001	Dual-Imp ⋆
GPT2-Small	+3.8%	3.25	0.004	11.59	< 0.001	Dual-Imp
GPT2-Medium	−8.7%	−7.91	< 0.001	10.00	< 0.001	Tradeoff
ERNIE 2.0	+2.1%	1.46	0.161	12.04	< 0.001	Utility-Dom
Genomic Foundation Models						
DNABERT-Base	−9.8%	−7.72	< 0.001	9.55	< 0.001	Tradeoff
DNABERT-2	−4.2%	−3.04	0.007	11.27	< 0.001	Tradeoff
DNAGPT	+10.5%	9.63	< 0.001	15.68	< 0.001	Dual-Imp
Nucleotide Trans.	+6.7%	5.63	< 0.001	12.53	< 0.001	Dual-Imp

Configuration Types: Dual-Imp = Dual Improvements (both privacy and utility significantly improve); Utility-Dom = Utility Dominant (privacy non-significant); Tradeoff = Privacy-Utility Tradeoff (utility improves, privacy degrades significantly).
⋆ indicates champion model (best overall performance).

References

[1] Reem Al-Saidi, Erman Ayday, and Ziad Kobti. Comparing reconstruction attacks on pretrained versus full fine-tuned large language model embeddings on homo sapiens splice sites genomic data. *arXiv preprint arXiv:2511.07481*, 2025. Accepted to IEEE TrustCom 2025.

[2] Xudong Pan, Mi Zhang, Shouling Ji, and Min Yang. Privacy risks of general-purpose language models. In *2020 IEEE Symposium on Security and Privacy (SP)*, pages 1314–1331, 2020.
